# In situ amplification of spin echoes within a kinetic inductance parametric amplifier

**DOI:** 10.1126/sciadv.adg1593

**Published:** 2023-03-10

**Authors:** Wyatt Vine, Mykhailo Savytskyi, Arjen Vaartjes, Anders Kringhøj, Daniel Parker, James Slack-Smith, Thomas Schenkel, Klaus Mølmer, Jeffrey C. McCallum, Brett C. Johnson, Andrea Morello, Jarryd J. Pla

**Affiliations:** ^1^ School of Electrical Engineering and Telecommunications, UNSW Sydney, Sydney, NSW 2052, Australia.; ^2^ Accelerator Technology and Applied Physics Division, Lawrence Berkeley National Laboratory, Berkeley, CA 94720, USA.; ^3^ Niels Bohr Institute, University of Copenhagen, Blegdamsvej 17, DK-2100 Copenhagen, Denmark.; ^4^ School of Physics, University of Melbourne, Melbourne, VIC 3010, Australia.; ^5^ School of Science, RMIT University, VIC 3001, Australia.

## Abstract

The use of superconducting microresonators together with quantum-limited Josephson parametric amplifiers has enhanced the sensitivity of pulsed electron spin resonance (ESR) measurements by more than four orders of magnitude. So far, the microwave resonators and amplifiers have been designed as separate components due to the incompatibility of Josephson junction–based devices with magnetic fields. This has produced complex spectrometers and raised technical barriers toward adoption of the technique. Here, we circumvent this issue by coupling an ensemble of spins directly to a weakly nonlinear and magnetic field–resilient superconducting microwave resonator. We perform pulsed ESR measurements with a 1-pL mode volume containing 6 × 10^7^ spins and amplify the resulting signals within the device. When considering only those spins that contribute to the detected signals, we find a sensitivity of $2.8\times 10^{3}\mathrm{spins}/\sqrt{\mathrm{Hz}}$ for a Hahn echo sequence at a temperature of 400 mK. In situ amplification is demonstrated at fields up to 254 mT, highlighting the technique’s potential for application under conventional ESR operating conditions.

## Teaser

High-sensitivity detection of spins has been achieved by performing amplification of spin signals within a superconducting device.

## INTRODUCTION

Electron spin resonance (ESR) spectroscopy is a technique used throughout physics, biology, chemistry, and medicine to study materials through their paramagnetic properties (*1*). To detect ESR, conventional spectrometers use a cavity to capture the weak microwave signal that is induced by the transverse magnetization of an ensemble of spins precessing in an external magnetic field. The magnitude of this signal is determined by the number of resonant spins coupled to the cavity (*N*), the spin-cavity coupling strength (*g*_0_), and the quality factor (*Q*) of the cavity. The coupling strength *g*_0_ depends on the degree of confinement of the magnetic energy in the microwave mode, $g_{0}\propto 1/\sqrt{V_{m}}$, where *V*_m_ is the magnetic mode volume (*2*). Conventional ESR spectrometers use three-dimensional (3D) microwave cavities where *V*_m_ ∝ λ^3^, with λ the wavelength of the resonant mode. An alternative approach is to use microresonator circuits, where the modes are confined in quasi-1D structures, such that *V*_m_ ≪ λ^3^ and *g*_0_ is considerably enhanced (*3*, *4*). Constructing these microresonator circuits from superconducting materials also allows them to achieve high-quality factors, which further enhances the spin sensitivity (*3*, *5*).

The superconducting circuit resonator is just one tool from the field of circuit quantum electrodynamics that has recently been applied to ESR. Josephson parametric amplifiers (JPAs) (*6*) have also been integrated into custom ESR spectrometers and used to push detection sensitivities to the quantum limit (*7*, *8*), where the noise in the measurement of a spin ensemble is set by vacuum fluctuations of the electromagnetic field. The combination of high-*Q* superconducting microwave resonators and JPAs has ultimately resulted in sensitivities as low as 120 spins for a single Hahn echo measured at 10 mK, corresponding to an absolute sensitivity of 12 spins/$\sqrt{\mathrm{Hz}}$ (*9*).

Another approach taken to improve ESR measurement sensitivity has been to couple the spin ensemble directly to nonlinear circuits. An early example of this involved coupling an ensemble of nitrogen vacancy centers in diamond to a superconducting transmon qubit, achieving a sensitivity of 10^5^ spins/$\sqrt{\mathrm{Hz}}$ (*10*). This was recently improved to 20 spins/$\sqrt{\mathrm{Hz}}$ using a flux-qubit read outvia an on-chip Josephson bifurcation amplifier (*11*). However, both of these approaches operate in a mode more analogous to continuous wave ESR spectroscopy. The efforts also mirror recent directions in the superconducting qubit community to engineer high-efficiency measurements by reducing or eliminating the insertion loss between the system being measured and the first cryogenic amplifier (*12*, *13*).

Despite these successes, previous works have all relied on technologies using Josephson junctions, which are not magnetic field resilient. This restricts the use of on-chip detection methods to low magnetic fields. Higher magnetic fields can be applied when using JPAs off-chip, where they are contained in magnetically shielded boxes and connected to the ESR cavity via coaxial cables and microwave circulators (*7*–*9*). The disadvantage of this approach is that the extra components introduce additional insertion loss, which inevitably attenuates the spin signals before they are amplified. Moreover, JPAs typically display gain compression for input powers ≳ − 110 dBm (*14*), which leads to signal distortion and limits the power that can be detected in pulsed ESR experiments.

In this work, we demonstrate that these limitations can be overcome by using a kinetic inductance parametric amplifier (KIPA) (*15*) coupled directly to an ensemble of spins. The KIPA is a weakly nonlinear microresonator engineered from the high kinetic inductance superconductor niobium titanium nitride (NbTiN), which is a material known to produce high-*Q* and magnetic field–resilient resonators (*16*, *17*). The weak nonlinearity of the KIPA allows it to act as both the ESR cavity and first-stage amplifier, where spin echo signals are amplified in situ via three-wave mixing (3WM) with an applied pump tone. Compared to using a low-noise cryogenic transistor amplifier as the first-stage amplifier, we demonstrate an enhancement of the signal-to-noise (SNR) ratio of 7.5 at a magnetic field of 6.8 mT and 3.8 at 250 mT, corresponding to a more than order of magnitude reduction in measurement times. Last, we discuss the excitation and detection bandwidths of our technique and explore a newly proposed amplification mode for 3WM devices, known as the Bogoliubov amplifier (BA), which exhibits an unrestricted gain-bandwidth product (GBP) and promises exciting features such as squeezing and noiseless amplification that make it an exciting tool for ESR spectroscopy.

## RESULTS

### Device design and characterization

**Fig. 1.**
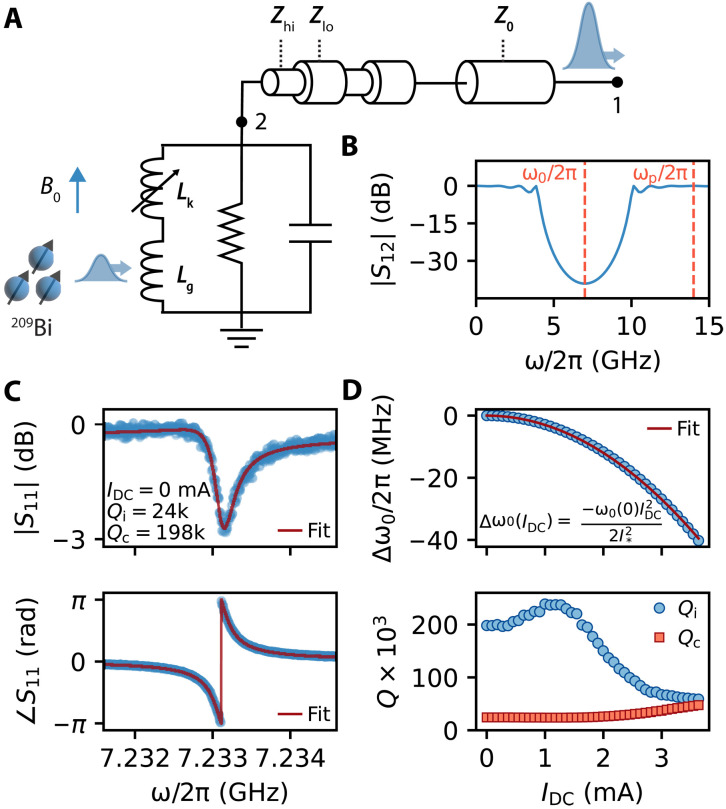
Device design and resonator characterization. (**A**) Schematic for the device. The resonator is depicted as a lumped element resonator with a geometric inductance (*L*_g_) that couples to an ensemble of ^209^Bi and a nonlinear inductance *L*_k_, which we exploit for amplification. The resonant mode is confined using a SIF, which we depict as a series of waveguides with alternating *Z*_hi_ and *Z*_lo_. (**B**) Frequency-dependent transmission of the SIF calculated from ABCD matrices. Note that port 2 is used here for illustration and is not physical. The frequencies of the resonator and pump tones are indicated. (**C**) Frequency-dependent magnitude (top) and phase (bottom) response of the device when measured in reflection (*S*_11_) with a vector network analyzer. The solid red lines correspond to a fit of the data in the complex plane, from which we extract the resonator’s parameters. (**D**) Shift in resonance frequency (top) and variation of the internal and coupling quality factors (bottom) of the device extracted from measurements of *S*_11_ made as a function of *I*_DC_. The solid line in the top panel is a fit to the equation in the inset.

The device used in this work is based on a KIPA (*15*) and consists of a λ/4 coplanar waveguide (CPW) resonator positioned at the end of a stepped impedance filter (SIF), as depicted in Fig. 1A. The SIF is constructed from a series of eight λ/4 impedance transformers with alternating high-(*Z*_hi_) and low-impedance (*Z*_lo_) (see the Supplementary Materials for detailed design specifications). The filter is analogous to an optical Bragg mirror and has been used to create Purcell filters for superconducting qubits (*18*) and 1D photonic crystals (*19*). We use the SIF to confine the resonator’s mode while maintaining a galvanic connection to it; this enables the resonance frequency (ω_0_) to be tuned by a DC current (*I*_DC_) (*20*–*22*) and facilitates amplification via 3WM when a pump with frequency ω_p_ ≈ 2ω_0_ is introduced (*15*, *23*). The predicted frequency-dependent transmission of a SIF can be calculated from ABCD matrices (*24*) and is plotted according to our design in Fig. 1B; it shows a deep stopband centered on ω_0_, which serves to isolate the resonant mode from the measurement port. Notably, the filter has passbands at DC and at ω_p_, so that both *I*_DC_ and the pump can be efficiently passed to the resonator.

The device is fabricated from a 50-nm-thick film of NbTiN on an isotopically enriched ^28^Si substrate that has been ion implanted with bismuth donors at a concentration of 1 × 10^17^ cm^−3^ over a depth of approximately 1.25 μm. The width of the resonator (i.e., the final λ/4 segment) center conductor is 1 μm with 10-μm gaps to ground and is designed to have a fundamental resonance at ω_0_/2π = 7.3 GHz with an impedance of *Z*_r_(ω_0_) = 240 Ω. The film exhibits a large kinetic inductance due to the inertia of the Cooper pairs, which displays a weak nonlinear dependence on the total current *I* according to (*25*)

$$L_{k}(I)=L_{k0}(1+I^{2}/I_{*}^{2})$$ (1)

where *L*_k0_ is the per-unit-length kinetic inductance at zero current of the NbTiN film (see the Supplementary Materials for details) and I_*_ is a constant related to the critical current (*15*). This nonlinearity allows the resonant frequency to be tuned through the application of a DC current *I*_DC_ and facilitates amplification. The spins couple to the magnetic field produced by the device or, equivalently, its geometric inductance *L*_g_. It is therefore important to balance the amount of kinetic and geometric inductance present to ensure a sufficient nonlinearity for performing amplification without substantially reducing the coupling to the spins (a detailed discussion on this is presented in the Supplementary Materials).

We use a vector network analyzer (VNA) to measure the response of the device in reflection (*S*_11_) without amplification and determine the experimental value of ω_0_/2π to be 7.233 GHz at *I*_DC_ = 0 mA (see Fig. 1C for the reflection magnitude and phase data). We also determine the internal quality factor (*Q*_i_) and coupling quality factor (*Q*_c_) by fitting the combined magnitude and phase response (*26*) and display the *I*_DC_ dependence of the fit results in Fig. 1D. The device can be tuned over a range of 40 MHz by applying up to *I*_DC_ = 3.63 mA before the superconducting film transitions to the normal state. We expect a quadratic dependence of the change in resonance frequency with the applied DC $\text{Δ}\omega_{0}(I_{\mathrm{DC}})=-\omega_{0}(0)I_{\mathrm{DC}}^{2}/(2I_{*}^{2})$, which allows us to extract I_*_ = 34.5 mA for this device. *Q*_i_ decreases monotonically for *I*_DC_ > 1.3 mA, and *Q*_c_ grows by a factor of two; the combined effect is that the device approaches critical coupling (*Q*_i_ = *Q*_c_) as *I*_DC_ is raised.

### Pulsed ESR measurements

**Fig. 2.**
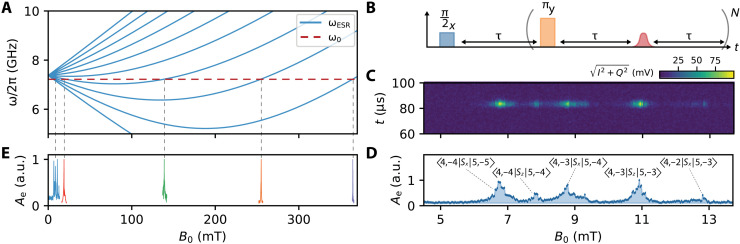
ESR measurements of^209^Bi donors in Si using a KIPA. (**A**) Allowed ESR transition frequencies for ^209^Bi in Si as a function of *B*_0_. We can measure ESR with the KIPA when ω_ESR_ = ω_0_, with the crossing points marked by vertical dashed lines. (**B**) CPMG sequence applied to detect the spins. We use τ = 75 μs and *N* = 200, averaging the echo produced by each of the *N* refocusing pulses to increase the SNR. (**C**) Homodyne-demodulated signal in the time domain as a function of *B*_0_, measured with the CPMG sequence shown in (B). The bright features correspond to a spin echo signal. (**D**) Integrated spin echo signal from (C). We label the five peaks according to the ESR transitions we expect from calculations of the spin Hamiltonian. a.u., arbitrary units. (**E**) Measurements of the *S_x_* transitions between 0 and 370 mT. Each measurement is independently normalized.

Our measurements are performed at a temperature of 400 mK, where the ^209^Bi donors bind one additional valence electron compared to the surrounding Si atoms. The donor-bound electron and its nucleus are coupled via the contact hyperfine interaction, *H_A_*/ℏ = *A***S** · **I**, where *A*/2π = 1.478 GHz (*27*) and **S** (**I**) represents the electron (nuclear) spin operator. At fields *B*_0_ < 100 mT, the contact hyperfine interaction is of comparable strength to the Zeeman interaction, *H_B_*/ℏ = *B*_0_(γ_e_*S*_z_ + γ_n_*I*_z_), where γ_e_/2π = 27.997 GHz/T and γ_n_/2π = 6.96 MHz/T. Here, the eigenstates of the spin Hamiltonian *H* = *H_A_* + *H_B_* are best described by the total spin **F** = **S** + **I** and its projection onto *B*_0_, *m_F_*. The 20 electron-nuclear states ∣*F*, *m_F_*〉 are divided into an upper manifold of 11 states with *F* = 5 and a lower manifold of nine states with *F* = 4. The ESR-allowed transitions, which are driven through the *S_x_* spin operator, are calculated and displayed in Fig. 2A, where we plot the frequency of the transitions as a function of the magnetic field.

The static magnetic field *B*_0_ is applied in the plane of the device and parallel to the long axis of the resonator. This alignment is chosen so that the magnetic field *B*_1_ produced by the λ/4 resonator, which drives spin resonance, is perpendicular to *B*_0_ for the spins located underneath the resonator to probe the *S_x_* ESR transitions. To perform ESR, we tune *B*_0_ so that ω_ESR_ = ω_0_ (horizontal dashed line in Fig. 2A).

We initially detect the spins without in situ amplification using a Carr-Purcell-Meiboom-Gill (CPMG) pulse sequence (Fig. 2B). Because of the long spin coherence times of ^209^Bi donors in isotopically enriched ^28^Si (*28*), we are able to repeatedly refocus the ensemble, collecting a spin echo each time we do so. We use *N* = 200 refocusing pulses and average the echo signals to increase the SNR ratio of our measurement (*29*). The result over a small field range is shown in Fig. 2C, where we plot the amplitude of the homodyne-demodulated signal, $\sqrt{I{\left(t\right)}^{2}+Q{\left(t\right)}^{2}}$, as a function of *B*_0_. In Fig. 2D, we also plot the integrated echo signal $A_{e}=(1/T_{e})\int_{0}^{T_{e}}\sqrt{I{\left(t\right)}^{2}+Q{\left(t\right)}^{2}}dt$, where *T*_e_ is the duration of the spin echo signal. By comparing the measured spectrum to an exact diagonalization of the Hamiltonian *H*, we can identify the transitions present in this field sweep. Three of the transitions are coupled by the *S_x_* operator. These are the typical ESR transitions that were expected for the orientation of the resonator and the external magnetic field. Two of the transitions, however, are coupled by the *S*_z_ spin operator. In the *F*, *m_F_* basis, these correspond to transitions of the type ∣4, *m_F_*〉 ↔ ∣5, *m_F_*〉 and are driven by a longitudinal magnetic field (i.e., when *B*_1_ is parallel to *B*_0_). Although they are forbidden at high field, these transitions can be observed at low field due to the hyperfine coupling, *H_A_* (*30*). This suggests that the resonant mode of our device is not fully confined to the last λ/4 section. Finite element simulations confirm this (see the Supplementary Materials) and show that at ω_0_, there is an appreciable *B*_1_ field in the last *Z*_hi_ segment of the SIF, which is oriented perpendicular to the resonator (see the Supplementary Materials for a full device schematic) and where *B*_1_ ∥ *B*_0_ for spins located underneath the inner conductor of the CPW. See the Supplementary Materials for a discussion on optimizing the mode distribution for performing ESR.

In addition to the five transitions measured at *B*_0_ < 13 mT, we demonstrate the ability to measure each *S_x_* transition out to 370 mT (Fig. 2E). Aside from the use of NbTiN as the superconducting material and the in-plane alignment of *B*_0_, we did not implement any specific features to enhance our device’s compatibility with magnetic fields, such as the inclusion of vortex pinning sites (*17*). By converting the magnetic field to the equivalent ESR frequency of a free electron, we see that this range of fields encompasses the X-band frequency range (8 to 10 GHz) common for commercial ESR spectrometers.

### In situ amplification of spin echoes

We now seek to use the weak nonlinearity of the NbTiN film to perform in situ amplification of the spin echo signals arising from standard Hahn echo pulse sequences. A pump tone is applied to the device at a frequency of ω_p_ and amplification occurs as energy transfers from the pump to the spin echo signal at ω_0_. The quadratic dependence of the kinetic inductance with current (see Eq. 1) naturally lends itself to a four-wave mixing (4WM) process, where energy conservation requires 2ω_p_ = ω_0_ + ω_i_, with ω*_i_* corresponding to the “idler” frequency—a tone produced during amplification. The application of a DC current lowers the order of the nonlinearity (*15*, *23*, *31*) and enables 3WM processes, where energy conservation dictates ω_p_ = ω_0_ + ω_i_. 3WM is preferable in amplification as it provides a large spectral separation between the pump and signal, allowing the pump to be filtered out of the detection chain. In addition, for kinetic inductance amplifiers, 3WM is a more efficient process than 4WM, requiring lower pump powers for equivalent gains (*23*).

**Fig. 3.**
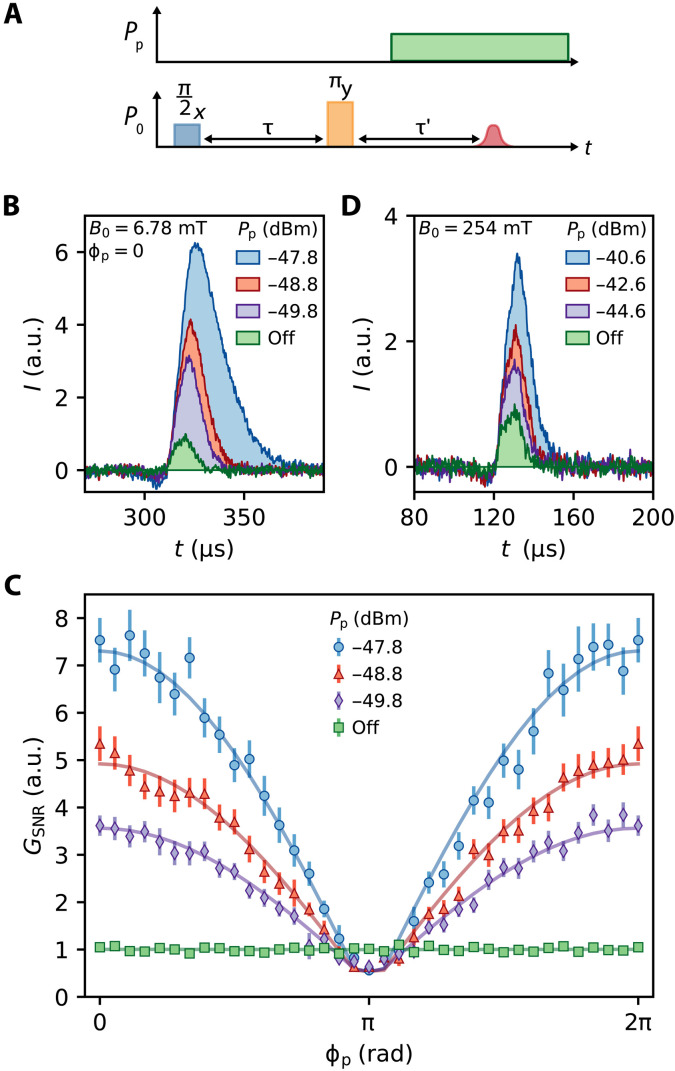
Degenerate amplification of spin echoes. (**A**) A modified Hahn echo pulse sequence where a strong parametric pump at frequency ω_p_ = 2ω_0_ and power *P*_p_ is supplied following the refocusing pulse. The device functions as a typical high-*Q* resonator for the first half of the pulse sequence and as a degenerate parametric amplifier during the period the spins induce a signal in the device. (**B**) Amplified spin echoes measured along the *I*-quadrature for several *P*_p_. For these measurements, ϕ_p_ = 0, *I*_DC_ = 3.0 mA, and *B*_0_ = 6.78 mT. The data are normalized to the measurement with the pump off. (**C**) *G*_SNR_ measured at the same set point as in (B). The improvement to the SNR is ϕ_p_ dependent because the amplifier is operated in degenerate mode. The error bars correspond to the SEM, and the solid lines are guides to the eye. (**D**) Amplified spin echoes measured with *I*_DC_ = 2.0 mA and *B*_0_ = 254 mT. Note that at this set point, we average measurements over the pump phase ϕ_p_. The data are normalized to the measurement with the pump off.

To amplify the spin echoes in situ via 3WM, we first DC bias the device with *I*_DC_ = 3.0 mA. Next, we apply a standard Hahn echo pulse sequence with the addition of a pump tone at the frequency ω_p_ = 2ω_0_ and power *P*_p_, which is sent 50 μs following the trailing edge of the refocusing pulse (Fig. 3A). The device therefore functions as a resonator during the delivery of the spin control pulses and as a resonant parametric amplifier when the spin echoes are detected. See the “Amplification timing and recovery” section in Materials and Methods for considerations regarding timing of the pump pulse. For a pump frequency at precisely twice the signal frequency (as used here), the signal and idler tones become degenerate and the gain depends on the relative phase between the signal and pump (*15*). In Fig. 3B, we compare spin echoes measured at *B*_0_ = 6.78 mT on the 〈4, −4∣*S_x_*∣5, −5〉 transition. The echoes are aligned along the *I* signal quadrature and presented for several different *P*_p_. The pump phase ϕ_p_ is tuned at each *P*_p_ to maximize the gain and therefore the spin echo amplitude. For *P*_p_ = −47.8 dBm, the maximum echo amplitude is greater than 7 times larger than that of an equivalent measurement with the pump turned off, where the first-stage amplifier corresponds to a cryogenic low noise high electron mobility transistor (HEMT) amplifier (see the Supplementary Materials for setup details). The duration of the echo also lengthens when *P*_p_ is increased. We attribute this to the finite GBP of the KIPA that is common to many resonant parametric amplifiers, which in our measurement extends the length of time it takes for the amplified intracavity field to decay.

### Signal-to-noise ratio

We define the amplitude SNR of the Hahn echo measurements as

$$\mathrm{SNR}=\frac{{\frac{1}{T_{e}}\int_{0}^{T_{e}}I(t)dt|}_{e}}{{\sqrt{\frac{1}{T_{e}}\int_{0}^{T_{e}}I^{2}(t)dt}|}_{b}},$$ (2)

where the *e* and *b* subscripts refer to the experimental pulse sequence (which produces a spin echo) and a blank pulse sequence (which gives a measure of the noise), respectively. For the blank sequence, we omit the π*_y_* refocusing pulse from the Hahn echo sequence so that no spin echo is produced. The amplitude SNR is therefore the ratio of the mean amplitude of the spin echo and the root mean square of the noise.

In our experiments, the duration of the spin echo depends on *P*_p_, so to calculate the SNR, we keep the window fixed to the duration of the shortest echo, corresponding to the measurement with the pump off (e.g., *T*_e_ ∈ [310 to 330] μs in Fig. 3B). This ensures that the bandwidth of the noise is equal when we compare the SNR for measurements taken with different *P*_p_. The improvement to the amplitude SNR when parametrically pumping the device is then given by

$$G_{\mathrm{SNR}}=\frac{{\mathrm{SNR}|}_{\mathrm{pump}\;\mathrm{on}}}{{\mathrm{SNR}|}_{\mathrm{pump}\;\mathrm{off}}}$$ (3)

In Fig. 3C, we demonstrate that *G*_SNR_ is phase dependent, displaying a period of 2π with the pump phase ϕ_p_. This is evidence that the amplifier indeed acts in degenerate mode. For these measurements, we align the maximum amplitudes of the different traces at ϕ_p_ = 0, where we find *G*_SNR_(ϕ_p_ = 0) = 7.5 ± 0.5 at the highest pump power *P*_p_ = −47.8 dBm. At this power, we also measure *G*_SNR_(ϕ_p_ = π) = 0.6 ± 0.1 , demonstrating that the spin echo signal is deamplified for certain phases.

In Fig. 3D, we show similar measurements taken at *B*_0_ = 254 mT, corresponding to the 〈4, −3∣*S_x_*∣5, −2〉 spin transition. Because of a gradual decline of *Q_i_* as we increase *B*_0_, we choose to work with *I*_DC_ = 2.0 mA, where ω_0_/2π = 7.218 GHz, *Q*_i_ = 37.9 × 10^3^, and *Q*_c_ = 27.3 × 10^3^. At this field, we manage to enhance the amplitude of the spin echo signal by up to a factor of 3.4 relative to a measurement without the pump applied, corresponding to *G*_SNR_ = 3.8 ± 0.3. Notably, this enhancement is achieved without explicitly optimizing ϕ_p_ to achieve maximum gain. The SNR of the spin signal at this transition was smaller than at 6.78 mT, requiring us to increase the number of measurement averages. Because of the limited hold time of our pumped ^3^He cryostat, this prevented us from measuring *G*_SNR_ as a function of ϕ_p_. Instead, we kept the microwave sources phase locked but randomized ϕ_p_ between repetitions, such that the *G*_SNR_ we report is effectively the average of *G*_SNR_(ϕ_p_). This SNR enhancement could therefore be improved by a factor of 2 with appropriate choice of pump phase.

We attribute the lower SNR of the spin echoes without in situ amplification at 254 mT (as compared to 6.78 mT) to the slightly smaller *S_x_* matrix element at this transition (〈4, −3∣*S_x_*∣5, −2〉 = 0.37 versus 〈4, −4∣*S_x_*∣5, −5〉 = 0.47) and the reduced internal quality factor of the resonator at high fields. Operating in the overcoupled regime (where *Q*_i_/*Q*_c_ ≫ 1) is known to produce spin echoes with optimal SNR in pulsed ESR spectroscopy (*32*). Field-induced losses here lower the ratio *Q*_i_/*Q*_c_ and therefore the SNR. By reducing *Q*_c_, we could place the resonator further in the overcoupled regime and make the device less susceptible to magnetic field–dependent losses. Lowering *Q*_c_ would also increase the bandwidth, which as discussed in the “Bandwidth” section below is advantageous as it permits faster pulses and hence broader spectrum excitations to be applied to the spins. Furthermore, the inclusion of vortex pinning sites has been shown to greatly suppress magnetic field–induced losses (*17*) and could be used in future devices.

**Fig. 4.**
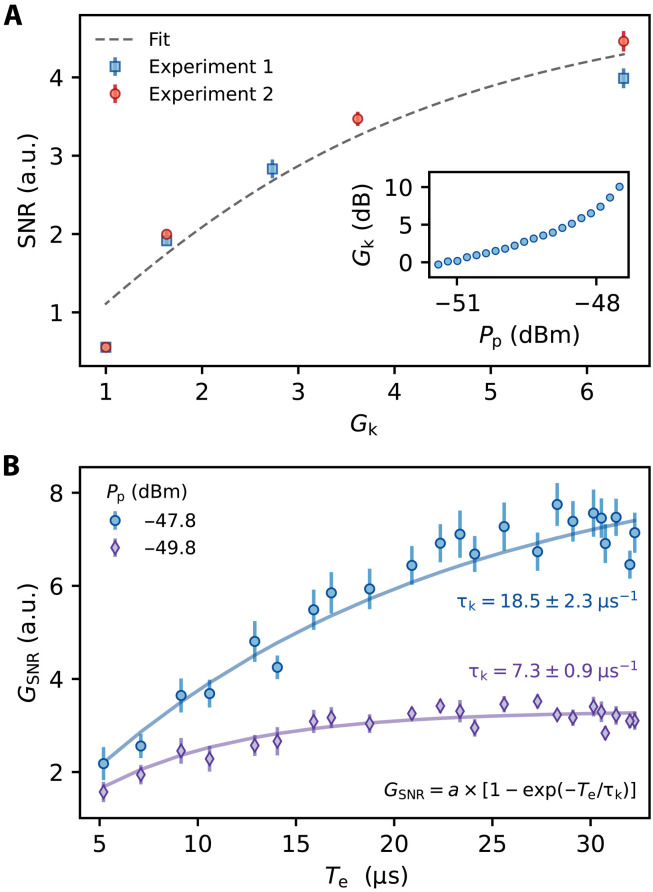
SNR gain and amplifier bandwidth. (**A**) SNR measured as a function of *G*_k_. The dashed line is a fit of the data to a model derived from cavity input-output theory (see the Supplementary Materials). Experiments 1 and 2 are equivalent experiments performed on different days. The data for experiment 2 were scaled by a factor of 1.33 so that the SNR with the pump off matches experiment 1. Inset: *G*_k_ measured as a function of *P*_p_. (**B**) *G*_SNR_ measured as a function of *T*_e_. The solid lines are fits to the equation in the inset.

Next, we investigate the dependence of *G*_SNR_ on the amplitude gain of the amplifier (*G*_k_; Fig. 4A). *G*_k_ is measured using a spectrum analyzer to assess the degenerate gain of a coherent signal with frequency ω_0_ reflected off the input of the KIPA and with ϕ_p_ chosen to maximize the gain (inset of Fig. 4A). We see that *G*_SNR_ initially grows rapidly with *G*_k_, but begins to saturate at high *G*_k_. This can be explained by considering the three contributions to the total noise: noise on the spin echo signal itself (*n*_s_), e.g., vacuum, thermal and spontaneous emission noise, noise added by the KIPA (*n*_k_), and noise added by the components following the KIPA (*n*_sys_). As we show using a model derived from cavity input-output theory in the Supplementary Materials, the SNR will grow with *G*_k_ so long as $G_{k}^{2}(n_{s}+n_{k})≲n_{s\mathrm{ys}}$. In our measurements, *n*_sys_ is dominated by insertion loss and the noise added by the cryogenic HEMT amplifier. The fit shown in Fig. 4A is based on our model and allows us to estimate the ratio of the system noise to the noise on the spin echo signal and the parametric amplifier added noise (see the Supplementary Materials). We find that *n*_sys_/(*n*_s_ + *n*_k_) ≈ 20, which agrees with our estimates for the system noise *n*_sys_ ≳ 12 photons per quadrature and for (*n*_s_ + *n*_k_) ≈ 0.6 photons per quadrature (see the Supplementary Materials). We note that we were not able to reach the high-gain limit in the present experiments. This occurs when *G*_k_ is large enough such that $G_{k}^{2}(n_{s}+n_{k})\gg n_{s\mathrm{ys}}$, whereafter *G*_SNR_ becomes independent of *G*_k_. When raising *G*_k_ beyond ∼6.5, we observed a hysteretic onset of parametric self-oscillations, which will be the focus of a future study.

### Bandwidth

The bandwidth of the pulses used to excite the spins is ultimately restricted by the spectral width of the resonator, which is κ_L_ = ω_0_/*Q*_L_ ≈ 2π × 0.26 MHz in the current device, where $Q_{L}={\left(Q_{i}^{-1}+Q_{c}^{-1}\right)}^{-1}$ is the loaded quality factor. This sets an effective cavity damping time of 2/κ_L_ = 1.2 μs. We note that the damping time can readily be decreased by reducing the *Z*_hi_/*Z*_lo_ impedance ratio in the SIF, and resonator bandwidths exceeding 10 MHz have already been demonstrated (*15*). This will permit the measurement of spin systems with short transverse relaxation times such as organic radicals and transition metal complexes (*33*), as are typically found in conventional ESR experiments. Furthermore, pulse shaping can be used to suppress cavity ring down and deliver shorter excitations to the spins, as demonstrated in previous superconducting microresonator studies (*34*).

The detection bandwidth is set by the in situ amplification bandwidth, which we noted earlier is governed by a constant GBP. The constant GBP is a feature of most resonant amplifiers, including the KIPA when operated in degenerate mode. Although as we show in the “The BA” section, it is also possible to use the KIPA in a regime that evades the constant GBP constraint.

To explore the effects of the constant GBP of the KIPA when operated as a degenerate parametric amplifier (DPA), we perform an experiment where we amplify the spin echoes while varying *T*_e_, which we achieve by varying the duration of the tipping and refocusing pulses in a Hahn echo sequence. For long *T*_e_, the bandwidth of the spin signal is smaller than the bandwidth of the KIPA and the entire echo signal is amplified. However, as *T*_e_ is made shorter, the bandwidth of the spin signal can exceed the amplification bandwidth of the device, which reduces *G*_SNR_. Because the device has a fixed GBP, we expect higher gains to produce smaller bandwidths. In Fig. 4B, we compare *G*_SNR_(*T_e_*) measured for two different *P*_p_ and find both experiments are well described by the function *G*_SNR_ = *a*[1 − exp(−*T*_e_/τ_k_)], where *a* and τ_k_ are constants. From the fits, we find *a*_1_/*a*_2_ = 2.7 ± 0.2 and τ_k1_/τ_k2_ = 2.5 ± 0.4, where the subscript 1(2) corresponds to the measurement with *P*_p_ = −47.8 dBm (−49.8 dBm). The close agreement of these two ratios confirms that the GBP is constant for the two experiments, because an increase in echo amplitude is associated with a corresponding increase in the echo duration required to saturate the SNR gain. We estimate the GBP from these measurements as GBP ≈ 0.8*a*/τ_k_ (see the “GBP estimation” section in Materials and Methods for a derivation of this relation), which is approximately 2π × 0.32 MHz for both pump powers.

### Spin sensitivity

The sensitivity of ESR measurements is often reported as the minimum number of spins (*N*_min_) required to achieve an SNR of unity for the detection of a single spin echo. By combining finite element modeling of the electromagnetic field distributions in the KIPA with the bismuth ion implantation profile, we estimate the total number of donors within the magnetic field of the resonant mode of the KIPA to be approximately 6 × 10^7^ (see the Supplementary Materials). Most of these donors do not contribute to the spin echo signals as they (i) do not populate the spin transitions probed, or (ii) are not excited by the selective pulses used in the sequence, or (iii) they do not rotate by the correct angles in the Hahn echo pulse sequence as a result of the inhomogeneous coupling of the spin ensemble to the resonator. See the Supplementary Materials for a detailed discussion and numerical estimates for each of these factors. Accounting for these effects, we estimate that the number of spins that contribute to the spin echo signals in our measurements is approximately 1 × 10^4^. Reporting the number of contributing spins is important as this figure does not depend on the sample being measured and is helpful for making comparisons with prior work (*7*–*9*).

Using the SNRs measured in Fig. 3, we find *N*_min_ ≈ 2 × 10^4^ contributing spins when the pump is off, which improves up to *N*_min_ ≈ 2.8 × 10^3^ by performing in situ amplification of the spin echoes. The absolute sensitivity, which takes into account the repetition rate of the measurement (1 Hz in our experiments), is thus found to be $2.8\times 10^{3}/\sqrt{\mathrm{Hz}}$. At the highest gain, this is equivalent to a concentration sensitivity of 4.2 nM when considering only the spins that contribute to the echo signals and 25 μM with regards to the total number of implanted spins. This compares well with a previous study at lower temperatures (*29*) that used a lumped element resonator and a JPA as a separate element in the detection chain. There the absolute spin sensitivity was found to be 1700 spins/$\sqrt{\mathrm{Hz}}$, i.e., within a factor of 2 of the result achieved here despite the current measurement operating at 400 mK with greater thermal noise (see the Supplementary Materials). We note that it is possible to boost the absolute spin sensitivity further by reducing the dimension of the resonator features (e.g., the width of the CPW inner track) to enhance the coupling to the spins, as has been demonstrated previously using submicron wires in lumped element resonators (*9*, *35*). Aside from improving sensitivity, scaling down the CPW track dimension would also reduce the critical current of the device and subsequently lower the pump power required for amplification (see the Supplementary Materials).

### The BA

The constant GBP restriction of a DPA causes the effective detection bandwidth to reduce as the gain is increased. To avoid artifacts associated with the narrowing bandwidth (such as time-stretched spin echoes), the bandwidth of the resonator, which dictates the GBP (*15*, *36*), should be made much larger than the desired detection bandwidth. An alternative approach is to operate the KIPA in a relatively unexplored regime recently proposed and implemented in the context of circuit quantum electrodyamics (*36*, *37*), which circumvents the fundamental constraint of a constant GBP. Here, instead of pumping the device at twice the frequency of the resonator mode, a frequency detuning much greater than the cavity linewidth is introduced: ω_p_ = 2ω_0_ − 2Δ, where ∣Δ∣ ≫ κ_L_/2. As the pump amplitude is increased, the system enters the Bogoliubov oscillator regime, with the emergence of signal and idler modes that exhibit gain over a fixed bandwidth set by the resonator linewidth κ_L_ (*36*). The device when operated in this mode is referred to as a Bogoliubov amplifier.

**Fig. 5.**
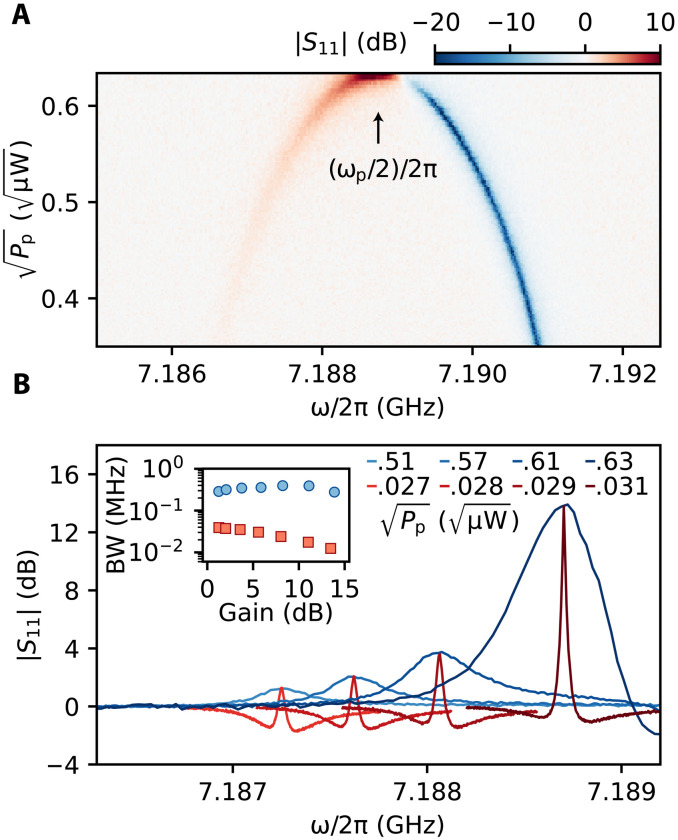
The BA and comparison with the resonant Δ=0 regime of operation. (**A**) Reflection magnitude response measured at different probe frequencies ω/2π for increasing pump amplitude $\sqrt{P_{p}}$ applied at ω_p_/2π = 14.3775 GHz (i.e., Δ/2π = 2.5 MHz). Data taken at *I*_DC_ = 3 mA. As $\sqrt{P_{p}}$ is increased, two features are observed, one below ω_p_/2 showing an increasing positive gain peak and another centered at the cavity resonance ω_0_ showing a dip, indicating that the resonator is operating close to critical coupling. The two features merge at high pump amplitude where the gain is maximum. (**B**) Selected traces at four different $\sqrt{P_{p}}$ for Δ/2π = 2.5 MHz (blue traces, ω_p_/2π = 14.3775 GHz) and Δ/2π = 0 (red traces, ω_p_ = 2ω_0_). The red traces are shifted by 2 to 4 MHz to align the amplification features with equivalent gain. Inset: Extracted bandwidths (BW) (plot on a logarithmic scale) for both amplification modes of operation in the main panel. Red squares correspond to the resonant (Δ = 0) configuration, and blue circles correspond to the BA. Additional data points are included in the inset that are not shown in the main panel. For gains larger than 5 dB, the amplification bandwidth can be enhanced by more than an order of magnitude when operated in the BA mode.

In Fig. 5A, we present ∣*S*_11_∣ reflection measurements of the KIPA in the presence of a pump tone with varying amplitude ($\propto \sqrt{P_{p}}$) and with a frequency that is detuned by 2Δ = 2π × 5 MHz from 2ω_0_. For large pump strengths, the signal and idler modes coalesce at the frequency ω_p_/2, as predicted by theory (*37*). Figure 5B depicts sample gain curves for the idler mode, which shows the gain rising as the pump amplitude is increased. For comparison, we also show gain curves for the KIPA operated under the same conditions as the in situ spin amplification measurements (Δ = 0), i.e., at degenerate parametric resonance ω_p_ = 2ω_0_. The traces at parametric resonance have been offset in frequency to align with the corresponding Bogoliubov plot of equivalent peak gain. Evident in these data is the significantly enhanced bandwidth of the BA. The inset in Fig. 5B shows the bandwidth of both amplifiers as a function of gain, where the BA bandwidth remains relatively constant, increasing slightly as the idler and signal modes coalesce, and the degenerate bandwidth reduces as the gain increases, corresponding to a constant GBP.

The BA has several intriguing properties that make it an exciting tool for ESR. As with the DPA, the BA is a quantum-limited amplifier that adds no noise in theory (*36*). Furthermore, the Bogoliubov oscillator is described by operators (β, β^†^) that correspond to squeezed bosonic operators (*a*, *a*^†^), such that β = cosh (*r*)*a* − sinh (*r*)*a*^†^ (with *r* the squeezing parameter) (see the Supplementary Materials for details). A remarkable consequence of the squeezed modes is that they can couple to other systems with an exponentially enhanced strength, as was demonstrated recently with a superconducting qubit (*37*) and using trapped ions (*38*). In ESR, we could therefore expect a coupling to the spins that grows as the gain of the BA is increased (see analysis in the Supplementary Materials), boosting the signal extracted from the spins and reducing the noise. While the squeezed modes are also expected to subject the spins to an increased dephasing (*39*), the large inhomogeneous broadening of most spin systems relative to typical spin-resonator coupling strengths (around 20 Hz in the current device; see the Supplementary Materials) implies that there is likely room for coupling enhancement before squeezing-induced dephasing becomes apparent.

## DISCUSSION

The enhancement observed in the spin sensitivity and SNR when amplifying with the KIPA can be attributed to an approximate 20 times reduction in the system noise temperature, which results from using a low-noise parametric amplifier and eliminating the insertion loss between our microresonator and amplifier. The success of this approach is evident when one compares the maximum *G*_SNR_ = 7.5 ± 0.5 to previous studies using JPAs separated from the microresonator and measured at 20 mK [*G*_SNR_ = 11.7 (*7*) and *G*_SNR_ = 5.9 (*8*)]; we achieve a similar improvement to the SNR in our measurements despite the fact that we operate at a higher temperature (400 mK) where the noise exceeds the quantum limit. The measurement SNR could be further improved in the future by optimizing the nonlinearity of the NbTiN film. Decreasing the amount of kinetic inductance relative to the geometric inductance would enhance the spin to resonator coupling strength and increase the total number of spins that contribute to the echo signals at the expense of requiring larger pump powers. We estimate that reducing the kinetic inductance fraction *L*_k_/(*L*_k_ + *L*_g_) from 0.8 in the present device to 0.4 would boost the SNR by a factor of 2 to 3 and require only a modest increase (<5 dB) in pump power to maintain the gains achieved here (see the Supplementary Materials for an analysis of the effect of kinetic inductance on SNR).

Future devices would benefit from a larger bandwidth to allow delivery of the short pulses that are needed to measure spin systems with small transverse relaxation times, as are typically found in ESR spectroscopy (*33*). The in situ amplification bandwidth can also be enhanced by operating the device as a BA, where we observed bandwidths more than an order of magnitude larger than that for the DPA at gains above 5 dB. The BA is also expected to display an exponentially enhanced coupling strength to the spins and therefore represents an exciting regime to study spin resonance in the future. We note that the enhanced coupling is only possible with in situ amplification as performed here, where the spins are directly coupled to the squeezed cavity mode.

Owing to the large critical temperature of NbTiN (*T*_c_ ∼ 13 K), the KIPA can readily be operated at higher temperatures (∼2 K), such as those accessible in pumped Helium-4 X-band ESR spectrometers. NbTiN kinetic inductance amplifiers have recently been shown to operate at temperatures up to 4 K (*40*). Combining high-temperature operation with the high magnetic field compatibility demonstrated here, this device could translate the high sensitivities achieved in bespoke quantum-limited ESR spectrometers to more conventional ESR operating conditions and systems.

The in situ detection of a small number of spins near a planar superconducting device opens exciting possibilities to probe exotic magnetic systems, such as molecule-based magnets. These chemically designed spins (*41*) offer interesting applications in quantum information processing (*42*), as their spin Hamiltonians can be tailored at the chemical level. These molecules can be functionalized to surfaces (*43*), which provides a pathway to introduce the spins into the small mode volume of the chip-based detector investigated here. For spin systems that cannot be functionalized to surfaces, microfluidic channels could be incorporated on the device to efficiently bring the sample within the resonator mode volume (*44*, *45*).

Last, we note that along with recent studies of 4WM parametric amplifiers made from NbN (*46*) and NbTiN (*47*), our demonstration here of a magnetic field–resilient superconducting parametric amplifier is promising not only for measurements of spin ensembles but also for a broad class of quantum experiments that combine magnetic fields with microwave measurements (*48*–*50*).

## MATERIALS AND METHODS

### Device and measurement setup

The device is fabricated from a 50-nm film of NbTiN on isotopically enriched ^28^Si (750 parts per million ^29^Si). The ^209^Bi donors are implanted uniformly across the entire substrate with a concentration of 10^17^ cm^−3^ over a depth of 1.25 μm. All measurements were performed at 400 mK using a pumped ^3^He cryostat and a homemade spectrometer. Further details on the device design and measurement setup are provided in the Supplementary Materials.

### Data processing

The measurements shown in Fig. 3 (B and C) were collected over an 18-hour period (approaching the maximum hold time for the pumped ^3^He cryostat). For each repetition of the experiment, the settings of *P*_p_ and ϕ_p_ were selected in pseudo-random order, with the control experiment for each setting performed immediately after each measurement. For each repetition, we recorded the average of 30 shots for each setting of *P*_p_, ϕ_p_, and their corresponding control sequences. A total of seven repetitions was completed so that the data shown in Fig. 3 (B and C) correspond to 210 shots total per data point. The pulse sequences were executed with a 1-Hz frequency, which is the same order of magnitude as 1/*T*_1_ (see the Supplementary Materials). The experiments collected in Fig. 3 (D and E) are composed of a total of 5000 shots of the pulse sequence, where ϕ_p_ was randomized for each measurement.

The SNR and *G*_SNR_ are calculated from post-processed data. From the raw data, we subtract a constant offset from both the *I* and *Q* quadratures, originating from components in the detection chain (amplifiers, mixers, digitizer, etc). We then downsample the data from the native 2−ns resolution of the digitizer to 50 ns and use a 1-MHz digital low-pass filter to reduce the noise. Next, we rotate the data in the *IQ*-plane such that the echo is aligned along the *I*-quadrature [by minimizing $\int_{t_{1}}^{t_{2}}|Q(t)|dt$]. For the phase-sensitive experiments, we correct for phase drift by fitting the normalized integrals *A_I_*(ϕ_p_) with the phenomenological function ∣sin (ϕ_p_ − π)∣. We note that the phase offsets acquired from the fits of the measurements with spin echoes are also applied to the corresponding control experiments to ensure that any imbalance in the gain of the *I* and *Q* detection channels is accounted for. The data shown in Fig. 3C correspond to the mean and SEM of the seven repetitions.

The data shown in Fig. 4A are from the same experiment as Fig. 3 (B and C), where ϕ_p_ = 0, and an equivalent measurement taken on another day. The SNR reported in Fig. 4A has been rescaled to that of a single shot as $\mathrm{SNR}=\bar{\mathrm{SNR}}/\sqrt{M}$, where $\bar{\mathrm{SNR}}$ is the SNR found from the mean of *M* shots of the Hahn echo pulse sequence. Experiment 1 (experiment 2) used *M* = 30 (*M* = 20) shots. The SNR for experiment 2 was found to be smaller than the experiment 1, which is likely due to a small drift in *B*_0_ or the resonator’s frequency. To account for this, we scale the data in Fig. 4A by a factor of 1.33 so that the SNR of experiment 2 with the pump off matches the SNR of experiment 1 with the pump off. The unscaled SNR and corresponding *N*_min_ for both experiments are summarized in the Supplementary Materials.

To extend *T*_e_ for the measurements shown in Fig. 4B, we varied the duration of the π/2 and π pulses in a Hahn echo sequence between 2 and 25 μs. *T*_e_ was then found using a threshold applied to the mean of the spin signal measured with the pump off.

### Amplification timing and recovery

In the in situ spin echo amplification experiments (see Fig. 3A), we apply the pump tone for a small window around the expected spin echo signal. Because the pump mode is not resonant (it is a traveling wave mode that lies within the wide passband of the stepped impedance filter), it can be switched on and off over short time scales. The typical pulse powers sent during the spin measurements are of order −70 dBm, and we wait for the resonator to ring down below 1% of its initial power after a pulse is applied before we activate the pump for amplification. Waiting several resonator damping times (i.e., 5 × 2/κ_L_ = 6 μs; see the “Bandwidth section” in Results) is sufficient for this purpose. We note that unlike transistor-based or even Josephson junction–based amplifiers, damage to the KIPA is highly unlikely if it is saturated (e.g., by the pulse ring down) because it does not contain any junctions that make it sensitive to electrostatic shock or breakdown. Upon turning the pump tone off, the amplified signal will ring down in a time once again set by the resonator bandwidth $\frac{2}{\kappa_{L}}=1.2\;\mu s$.

### GBP estimation

To extract an effective GBP from the measurement of the SNR gain versus echo duration (Fig. 4B), we first assume that the echo spectrum can be approximated by a Gaussian function

$$A_{e}(\omega )=A_{0}\exp[-\omega^{2}/(2\sigma^{2})],$$

where σ is the SD of the spectrum. In the experiment, we define the echo duration *T*_e_ as approximately four SDs of the time echo trace, such that σ = 4/*T*_e_. Next, we assume a Lorentzian profile for the in situ amplification gain of the form

$$G_{k}(\omega )=(a-1)\frac{{\left(\text{Γ}/2\right)}^{2}}{\omega^{2}+{\left(\text{Γ}/2\right)}^{2}}+1$$

where Γ is the full width at half maximum and *a* is the peak gain. If we assume that the change in the SNR gain with echo duration *T*_e_ is purely a result of the varying overlap of the spin echo signal spectrum with the amplifier gain profile, then we can estimate the 3-dB bandwidth of the amplifier from the point at which the SNR amplitude gain is reduced by factor of $\sqrt{2}$

$$\frac{\int_{-\infty }^{\infty }A_{e}(\omega )G_{k}(\omega )\;d\omega }{\int_{-\infty }^{\infty }A_{e}(\omega )\;d\omega }=\frac{a}{\sqrt{2}}$$

Solving this numerically, we find Γ/2π ≈ 1.23/τ_k_ in the high-gain limit (*a* ≫ 1), where τ_k_ is the time constant extracted from the data in Fig. 4B. This allows us to specify the bandwidth of the amplifier (defined as the width at an amplitude gain of $a/\sqrt{2}$) as BW/2π ≈ 0.8/τ_k_. Last, the GBP is then provided as the peak amplitude gain multiplied by the bandwidth GBP/2π ≈ 0.8*a*/τ_k_.

## Supplementary Materials

### This PDF file includes:

Materials and Methods Supplementary Text Figs. S1 to S8 Tables S1 to S4 References
