# Supplementary material for: In situ amplification of spin echoes within a kinetic inductance parametric amplifier
Source: Sci Adv. 2023 Mar 10;9(10):eadg1593. doi: 10.1126/sciadv.adg1593 (PMC10005168; doi:10.1126/sciadv.adg1593)
Supplement: Supplementary file 1 — Materials and Methods Supplementary Text Figs. S1 to S8 Tables S1 to S4 References [file sciadv.adg1593_sm.pdf]

Science Advances
AAAS

Supplementary Materials for

In situ amplification of spin echoes within a kinetic inductance parametric amplifier

Wyatt Vine *et al.*

Corresponding author: Jarryd J. Pla, jarryd@unsw.edu.au

Sci. Adv. **9**, eadg1593 (2023)
DOI: 10.1126/sciadv.adg1593

This PDF file includes:

Materials and Methods
Supplementary Text
Figs. S1 to S8
Tables S1 to S4
References

I. EXPERIMENTAL SETUP

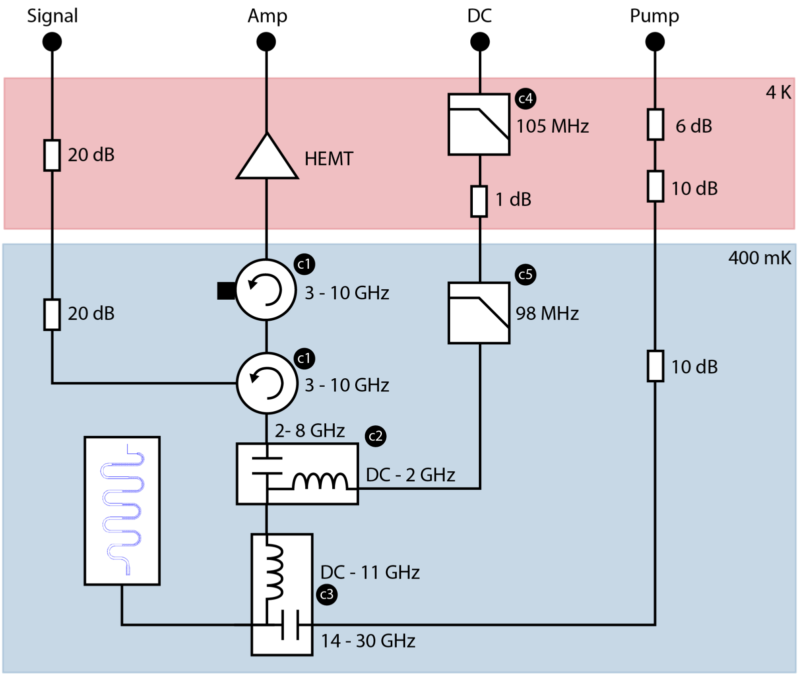

FIG. S1. The measurement setup. The components are (c1) Raditek RADC-4-10-Cryo circulator, (c2) Marki DPXN4, (c3) Marki DPX1114, (c4) Mini-Circuits VLF-105+, (c5) Mini-Circuits SLP-100+.

All measurements were performed at 400 mK using a pumped ^{3}He cryostat. A schematic of the measurement setup is shown in Fig. S1. It consists of four semi-rigid coax cables between room temperature and the ^{3}He stage of the cryostat. A description of each measurement line is provided below.

- The signal line was used to deliver resonant power to the device ($\omega_0/2\pi \approx 7.2$ GHz). It had a total of 40 dB of fixed attenuation, split across the two stages of the cryostat. We measure the attenuation from the room temperature input to the device to be -52 ± 1 dB at ω_0 when the system is cold (by combining $|S_{21}|$ measurements made across multiple cooldowns).

- The DC line was used to supply a DC current to the device and was low-passed filtered with a cut-off of 100 MHz.

- The pump line was used to supply high frequency microwave tones at $2\omega_0$. It had a total of 26 dB of fixed attenuation. The total attenuation at ω_0 is estimated to be more than 60 dB.

- The amplification line contains a single HEMT at the 4 K. It is connected to the signal line via a circulator and is isolated from the device via a second 50 Ω terminated circulator. The connection between 4 K and the amplifier is a superconducting NbTi coax cable, to reduce loss.

The general goal of this setup is to combine signals across the three distinct frequency ranges together at 400 mK so that the device can be measured via a single port. We accomplish this using a bias-tee to combine the DC and signal tones (Marki DPXN4) and a diplexer to further combine the pump (Marki DPX1114). In addition, each of the three lines is attenuated by at least 50 dB at ω_0, greatly surpassing the temperature difference between room temperature and the coldest stage which is $10\log_{10}(300\text{ K}/400\text{ mK}) \approx 30$ dB.

All of the pulsed ESR measurements were obtained with a home-built spectrometer depicted in Fig. S2. The system has two main arms: one for generating phase-controlled pulses, and one for performing homodyne demodulation of the reflected signals. Pulses are generated from a Local Oscillator (LO) with fixed power using two channels of an Arbitrary Waveform Generator

(AWG) with a microwave IQ-mixer. To extend the dynamic range of the system, a programmable attenuator can be used to vary the output power of the system. To ensure no unintended power leaks out of the box, a fast microwave switch is placed before the output of the system and is actuated throughout the pulse sequences. Similar switches are placed at the input of the box and provide 40 dB of attenuation when open to ensure that the high power pulses used for performing ESR do not reach the demodulator with a power exceeding its damage threshold. Power entering the box is further amplified before being homodyne demodulated. The resulting quadrature signals are digitized by a Data Acquisition System (DAQ). We route the pump through the box and use fast microwave switches to gate its output. Triggering of the AWG, DAQ, and microwave switches is achieved with TTL logic supplied by Spin-Core PulseBlaster ESR Pro.

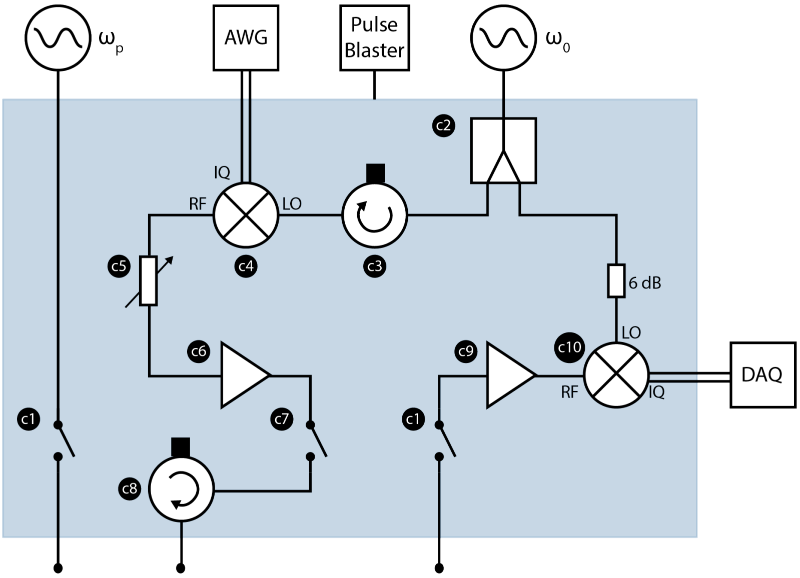

FIG. S2. Schematic for a home-built spectrometer for pulsed ESR measurements. c1: single-pole double-throw microwave switch, c2: Marki PD-0R510, c3: Ditom D3C4080, c4: Marki IQ-4509LXP, c5: Mini-Circuits RUDAT-13G-60, c6: Mini-Circuits ZVE 3W-183+, c7: double-pole quad-throw microwave switch, c8: Ditom D3C4080, c9: Mini-Circuits ZX60-05113LN+, c10: Polyphase AD60100B.

II. DEVICE DETAILS

A schematic of the device we use is shown in Fig. S3a. It consists of a $\lambda/4$ resonator formed from a CPW with centre track width $w = 1$ μm and gap width $g = 10$ μm, shorted to the ground plane on one end and galvanically connected to a Stepped Impedance Filter (SIF) on the other. It has a total of eight segments with alternating impedance ($4 \times Z_{\rm lo}$ and $4 \times Z_{\rm hi}$), each of which has an electrical length of $\lambda/4$ at ω_0. The dimensions of the CPWs used for each section of the device are summarized in Table S1. The B_0 field is nominally aligned in plane and parallel to the long axis of the resonator. In this configuration, the resonant current produces a B_1 field that is perpendicular to B_0 for ^{209}Bi located underneath the centre conductor of the resonator.

TABLE S1. Device design parameters.

	Bondpad	SIF $Z_{\rm lo}$	SIF $Z_{\rm hi}$	SIF $Z_{\rm hi}$ (final section)	Resonator
w (μm)	100	138	10	5	1
g (μm)	45	6	70	15	10

To fabricate the device we begin with a 15 μm thick epitaxial layer of ^{28}Si (with approximately 750 ppm residual ^{29}Si) grown on a 300 μm high-resistivity non-compensation-doped natural-silicon wafer (> 5 kΩ). We implant ^{209}Bi donors uniformly across the wafer with a rectangular implantation profile with a target concentration of 10^{17} cm^{-3} from 0.35 μm to 1.5 μm depth (Fig. S4).

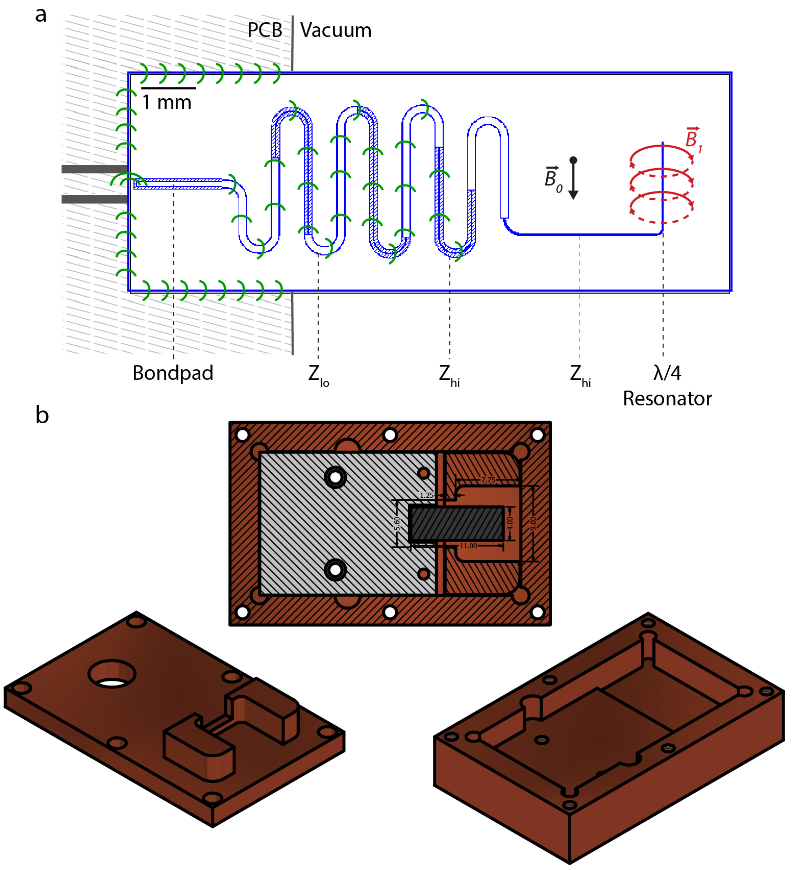

FIG. S3. Device and enclosure design. (a) A top-down view of the device. The white areas correspond to a thin film of NbTiN. The blue areas have been etched away to reveal the Si underneath. All of the components are fabricated from a continuous CPW with the dimensions specified in Table S1. The orientation of the static B_0 field is in plane and parallel to the long axis of the resonator. A layout of the wirebonds (solid green lines) similar to the one that was used is shown. (b) A top-down cross-sectional view of the enclosure and device assembly depicting the device (dark grey) and PCB (light grey) (top), the enclosure lid (bottom left), and the enclosure base (bottom right).

Damage to the Si lattice caused by the implantation is repaired via a 20 min anneal at 800 °C in a N_2 environment, which also aids in the incorporation of ^{209}Bi into the host crystal. It is expected that this procedure results in the electrical activation of approximately 60% of the implanted donors [51]. Following this, a 50 nm film of NbTiN is sputtered (Star Cryoelectronics). From independent calibrations we determine that the sheet kinetic inductance is $L_\square = 3.5$ pH/□, which is related to the per-unit-length kinetic inductance via $L_{k0} = L_\square / w$ for a thin-film trace of width w. The resonator is patterned with EBL and etched with a CF_4 and Ar plasma.

The device is mounted within a 4×3 mm^2 groove in a Printed Circuit Board (PCB) with wax. The PCB is made from 0.635 mm thick Rogers RO3006 laminate covered with 1 oz of copper on both sides with an immersion silver finish. We use wirebonds to connect the PCB and device ground. We also place wirebonds across each of the first six $\lambda/4$ segments of the SIF to prevent excitation of slotline modes (see Fig. S3a for a wirebond schematic similar to the one that was used). The device is placed in a gold-plated oxygen-free copper enclosure (Fig. S3b). The body and lid of the enclosure are machined so that the 8 mm length of the resonator that is not supported by the PCB extends within a small 3D cavity defined by the enclosure base and the machined lid. The chip enters the cavity through a small constriction that forms a rectangular waveguide with a cut-off frequency of ~ 27 GHz and the cavity is designed to support a fundamental resonant mode at ~ 45 GHz. Most of the SIF and the entire resonator are hosted within the cavity, which acts to suppresses radiation losses from the device.

III. ESTIMATING THE SENSITIVITY N_{min}

The sensitivity is defined as the minimum number of spins (N_{min}) required to produce a single spin echo with a SNR = 1. In order to determine N_{min} for our spectrometer, we measure an echo signal to find the single-shot SNR and combine this with an estimate of the total number of spins (N_{tot}) that contribute to the echo signal. In the following section we provide detailed steps on how to calculate N_{tot} for our device and provide an estimate for N_{min} for the experiments we performed at $B_0 = 6.78$ mT.

A. Implanted donors inside the magnetic mode volume

Bismuth donors have been implanted over the entire surface of the chip, with an implantation profile shown in Fig. S4. However, only donors that interact with the magnetic mode of the resonator contribute to the echo signal. As a simple approximation, we could assume that the mode is confined to the $\lambda/4$ segment at the end of the device (designed to be the resonator), which consists of a coplanar waveguide with an inner track width of $w = 1$ μm and a length of $l = 1.75$ mm. The implantation profile is approximately rectangular, with a concentration of $C_d = 1 \times 10^{17}$ cm^{-3} from a depth of 0.35 μm to 1.6 μm. This provides an effective 'donor volume' of $V_d \approx wl\Delta d = 2.2 \times 10^{-15}$ m^3 (where $\Delta d = 1.25$ μm is the implantation depth range) and a number of implanted donors $N_d = \beta_a C_d V_d = 1.3 \times 10^8$ able to interact with the resonator. This estimate accounts for the 60% bismuth electrical activation yield ($\beta_a = 0.6$) under the annealing conditions used here [51].

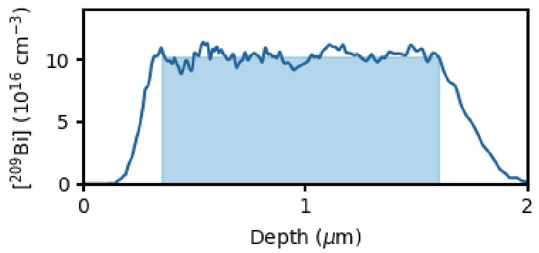

FIG. S4. Donor implantation profile. The concentration of ^{209}Bi as a function of depth, simulated with the software package TRIM. The spins were implanted uniformly across the entire substrate. The shaded box corresponds to the rectangular profile used in our calculation of N_{min}.

This crude estimate of N_d can be improved with detailed knowledge of the magnetic field distribution of the resonant mode in the device. This would allow us to calculate N_d as

$$\begin{aligned} N_d &= \frac{\int |B_{1\perp}(r)|^2 \rho(r) dV}{\max(|B_1|)^2}, \\ &\approx \beta_a C_d \frac{\int_{\mathrm{imp.}} |B_{1\perp}(r)|^2 dV}{\max(|B_1|)^2}, \\ &= \beta_a C_d V_d, \end{aligned} \tag{1}$$

where $\rho(r)$ is the implanted and activated bismuth donor concentration, $B_{1\perp}(r)$ is the component of the B_1 field perpendicular to the static field B_0 and $\max(|B_1|)$ is the maximum strength of the field over the whole mode. Using $B_{1\perp}(r)$ in the numerator of Eq. 1 ensures that we only consider spins in the device which can be driven through the $|F, m_f\rangle = |4, -4\rangle \rightarrow |5, -5\rangle$ transition that we probe at ~ 6.78 mT. In the second line we assume that $\rho(r) = \beta_a C_d$ is constant over the depth range $0.35 - 1.6$ μm and zero otherwise, with the integration volume being restricted to this implantation depth range. We define the effective donor volume to be

$$V_d = \frac{\int_{\text{imp.}} |B_{1\perp}(r)|^2 dV}{\max(|B_1|)^2} \quad (2)$$

$$= \underbrace{\left(\frac{\int |B_{1\perp}(r)|^2 dV}{\max(|B_1|)^2}\right)}_{V_m} \underbrace{\left(\frac{\int_{\text{imp.}} |B_{1\perp}(r)|^2 dV}{\int |B_{1\perp}(r)|^2 dV}\right)}_{\eta}, \quad (3)$$

which provides a more accurate measure than one based on simple geometric arguments presented above. In the second line we show that V_d can also be expressed as $V_d = V_m \eta$, where V_m is the total volume of the perpendicular magnetic field component (the magnetic mode volume) and η is the proportion of the perpendicular field occupied by the implanted spins (the spin filling factor).

To evaluate $B_1(r)$ we use CST Studio Suite to perform a finite-element simulation of the full planar device structure, incorporating kinetic inductance, bond wires and the device enclosure. We utilise the frequency domain solver to calculate the magnetic field profile of the fundamental resonator mode and re-scale the result to provide the root-mean-square (RMS) field fluctuations ($\delta B_{1\perp}(r)$). Calculating $\delta B_{1\perp}(r)$ has the dual purpose of allowing us to determine the single spin to resonator coupling strength (g_0), which we consider later in Sec. III B. We find $\delta B_{1\perp}(r)$ using the relation

$$\delta B_{1\perp}(r) = \frac{B_{1\perp}(r)}{2\sqrt{\bar{n}}}, \quad (4)$$

where $\bar{n}$ is the number of intracavity photons and is given by [52]

$$\bar{n} = \frac{4\kappa_L}{\hbar\omega_0(\kappa_L + \gamma)^2} P_{\text{in}}. \quad (5)$$

Here $P_{in} = 0.5$ W is the input power used in the simulation, ω_0 is the fundamental mode frequency, $\kappa_L = \omega_0/Q_L$ where Q_L is the loaded quality factor, and $\gamma = \omega_0/Q_i$, where Q_i is the internal quality factor. Fig. S5a and b depicts the $\delta B_{1\perp}(r)$ distribution of the fundamental resonator mode.

Evaluating Eq. 2 using our CST simulation result we find $V_d = 1$ pL, which using Eq. 1 leads to $N_d = 6 \times 10^7$. This value is smaller than the estimate derived from simple geometric arguments because the latter does not factor in the orientation of the B_1 field. This calculation is also more accurate because it takes into account the fact that the mode is not strictly confined to the last $\lambda/4$ segment, but instead penetrates into the stepped-impedance filter (see Fig. S5a,b). Table S2 shows the effective donor volumes for the different regions in the device, where we find that approximately half of the mode exists in the last segment of the SIF.

TABLE S2. Effective donor volumes throughout the device. 1 μm^3 = 1 fL.

Section	V_d (μm^3)
SIF (first sections)	189.5
SIF (final section)	549.8
Resonator	270.5
Total	1009.8

B. Coupling strength distribution

In microresonator devices the spin-photon coupling strength (g_0) is highly inhomogeneous due to the spatial distribution of the magnetic field. A spin echo measurement therefore only measures the subset of spins where the pulses provide well-calibrated π and $\pi/2$ rotations [53, 54]. We must therefore also estimate what fraction of spins have coupling strengths that contribute to the echo signals. We do this by measuring g_0 via a Rabi oscillation experiment and simulating the coupling strength distribution in our device, as detailed below.

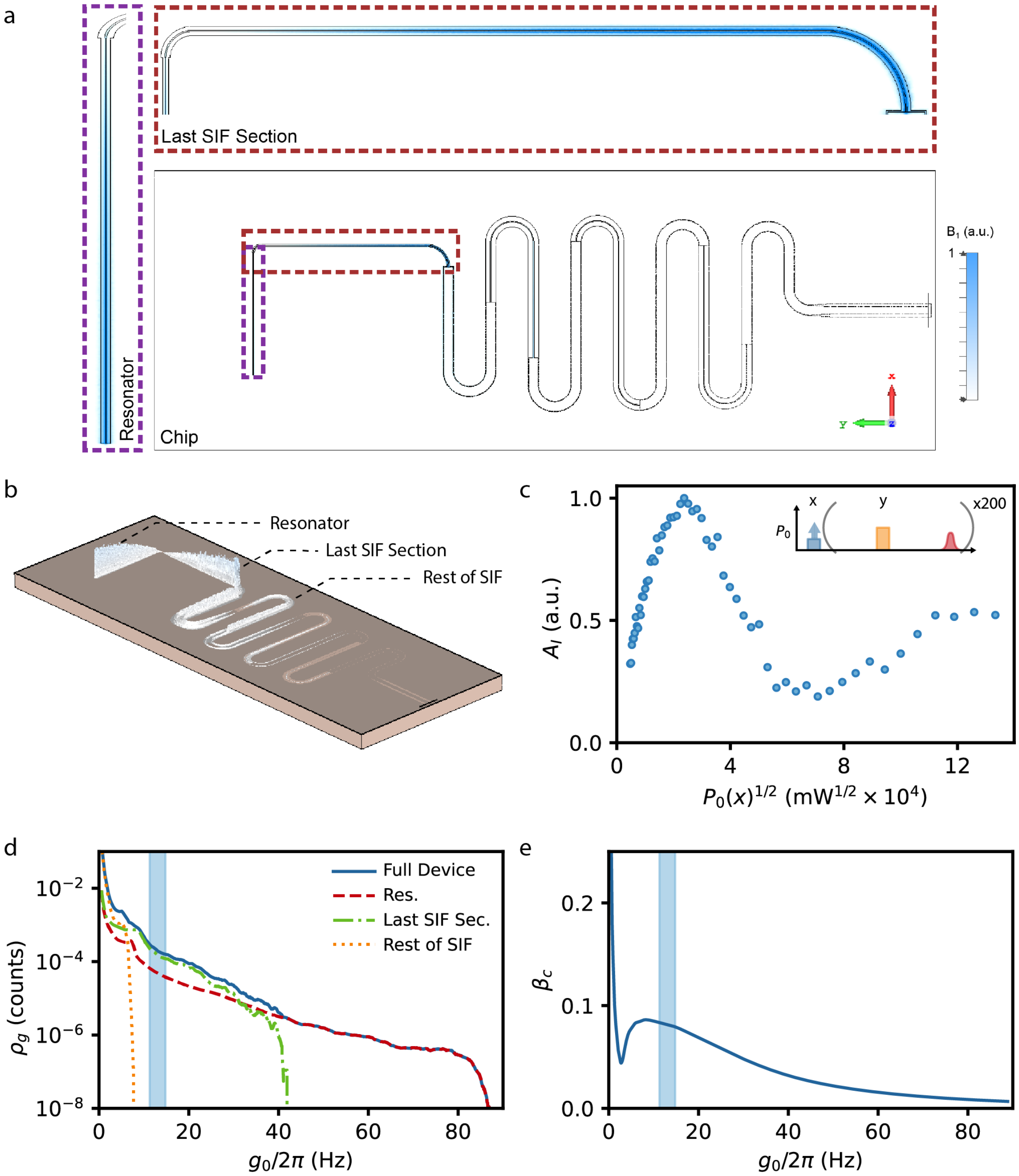

FIG. S5. Distributions of spin-photon couplings. (a) A finite element simulation of the resonant B_1 magnetic field perpendicular to the static field B_0, taken for a 2D slice beneath the resonator. (b) From the simulation in (a), the strength of the magnetic field at ω_0 is depicted as an out-of-plane gradient fill, which shows that the magnetic field is concentrated in the resonator and the last section of the SIF. (c) Rabi oscillations measured as a function of the tipping pulse power. Inset: a depiction of the CPMG-200 sequence used. (d) Normalized histograms depicting the number of total spins that couple to the magnetic mode of the device ($\propto \bar{V}_d$) as a function of g_0. (e) β_c, the ratio of total spins that contribute to a spin echo, as a function of g_0. In (d) and (e) the blue bars correspond to the range of g_0 extracted from the Rabi oscillations.

1. *Experiment*

We can extract an average g_0 for the spins experimentally by measuring the Rabi frequency (Ω_R) along with the average number of intracavity photons ($\bar{n}$) generated by the input pulse. They are

related by [2]:

$$\Omega_R = 2g_0\sqrt{\bar{n}}, \tag{6}$$

where $\bar{n}$ is calculated from Eq. 5.

In Fig. S5c we perform Rabi oscillations by fixing the durations of the π and $\pi/2$ pulses ($t_{\rm pulse}$ = 3 μs) in a CPMG-200 measurement sequence and varying the power of the $\pi/2$ pulse. We assign the power at which A_I is maximum to be the $P_0(\pi/2)$, which in this case was -73 ± 1 dBm, referred to the input of the device. This power is determined by subtracting the loss between the room temperature microwave source and the sample, which was calibrated by combining measurements made across a series of cool downs of our cyrostat. We estimate $\bar{n} = 1.00^{1.26}_{0.80} \times 10^7$ from VNA measurements of S_{11} measured at the same power as $P_0(\pi/2)$, where the upper and lower values correspond to the uncertainty in P_0. We therefore extract $g_0/2\pi = 13.2^{14.8}_{11.7}$ Hz.

2. *Simulation*

Our CST Suite simulation of $\delta B_{1\perp}$ (see Sec. III A) can be used to calculate the distribution of spin-resonator coupling strengths using the formula:

$$g_0 = \delta B_{1\perp} M \gamma_e, \tag{7}$$

where $M = 0.473$ is the matrix element for the $|F, m_f\rangle = |4, -4\rangle \rightarrow |5, -5\rangle$ transition at B_0 = 6.78 mT and $\gamma_e/2\pi$ = 28 GHz/T is the gyromagnetic ratio. In Fig. S5d we show a normalized histogram of the number of spins that couple to the resonant mode (ρ_g) as a function of g_0. Comparing the histograms for different parts of the device it becomes apparent that the SIF, which contains larger CPW dimensions than the rest of the structure, has the weakest coupling strengths. The last segment of the SIF and the resonator produce much larger couplings, due to the tighter confinement of the magnetic field in these regions. The range of g_0 corresponding to our experimental measurement are indicated by a blue bar, where the ratio of spins located underneath the last section of the SIF and the resonator is approximately 3 : 1.

3. *Spin fraction*

Using the simulated distribution of g_0 in our device and the experimentally extracted g_0, we can estimate the fraction of resonant spins that contribute to an echo. We define the spin fraction as [53]:

$$\beta_c(g_0) = \frac{\int g\rho_g(g)\sin^3\left(\frac{\pi g}{2g_0}\right)dg}{\int g\rho_g(g)dg}, \tag{8}$$

and plot $\beta_c(g_0)$ in Fig. S5e. Over the range of g_0 extracted from our measurement (indicated by a blue bar) we find $\beta_c = 0.082^{0.083}_{0.079}$.

C. Pulse excitation bandwidth

Because we use a high-Q resonator, we must also consider that the bandwidth of the spins we excite in pulsed measurements is smaller than the bandwidth of the ESR transitions. It is therefore necessary to find the overlap of the pulse excitation bandwidth with the spectral density of a given ESR transition to estimate the proportion of spins that are excited.

In Fig. S6a we measure a ^{209}Bi ESR transition centred at B_0 = 6.76 mT using a Carr-Purcell-Meiboom-Gill (CPMG) sequence with 200 refocusing π pulses as we sweep B_0. Previous investigations of ^{209}Bi ESR transitions in Si using high-Q aluminium resonators have found broad spin

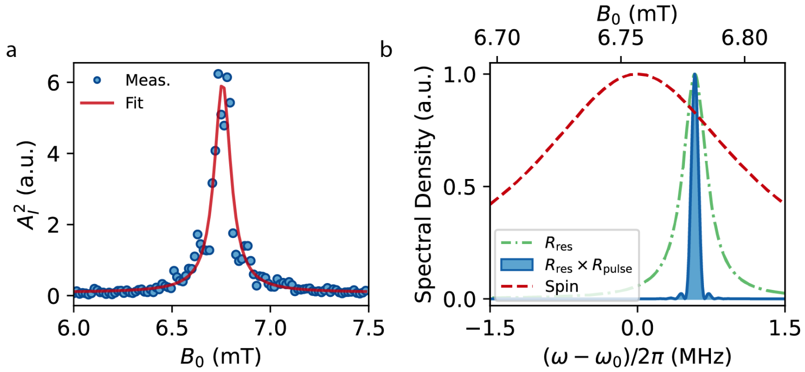

FIG. S6. Spin and resonator spectral density. (a) A measurement of the $|F, m_f\rangle = |4, -4\rangle \rightarrow |5, -5\rangle$ transition using a CPMG sequence with 200 π pulses. The data is fit with a Lorentzian from which we extract a linewidth of 0.10 mT. (b) The magnitude response of the cavity (dashed green line) and the cavity-filtered pulse power spectrum (blue fill) are considerably narrower than the spin linewidth (dashed red line).

distributions that were non-uniform with B_0 [7]. For those devices, the transitions were determined to be broadened by local strain, caused by the larger thermal contraction of the aluminium resonator compared to the silicon substrate [30, 55]. Since the ESR transitions we observe in our device are similarly broad, this strongly suggests a similar effect of local strain in our device. Here we estimate the ratio of spins that lie within the bandwidth of our measurement by comparing the spectral density of the spin transition with the power spectrum of the pulses employed in the measurements.

We define the spin spectral density as $A_I^2(B_0) = \int_{T_E} I^2 dt$, where T_E defines the spin echo duration. From numerical calculations of the ^{209}Bi spin Hamiltonian we determine that the ESR transition resonant with our device is the $|F, m_f\rangle = |4, -4\rangle \rightarrow |5, -5\rangle$ transition, which has $(\partial\omega/\partial B)/2\pi = -25.06$ MHz/mT. This allows us to convert A_I^2 from a function of B_0 to a function of $\omega - \omega_0$. We note that for a spin ensemble inhomogenously broadened by strain, there is no strict functional form expected for the lineshape [30, 55], but for this experiment a Lorentzian seems to fit the data well. This therefore allows us to estimate the spin linewidth to be 0.10 mT, or equivalently 2.55 MHz.

The bandwidth of spins that are excited by a pulse (R) is given by the product of the magnitude response of the resonator (R_{res}) and the pulse power spectrum (R_{pulse})

$$\begin{aligned} R_{\text{res}}(\omega) &= \frac{1}{1 + 4(\omega - \omega_0)^2/\kappa_L^2}, \\ R_{\text{pulse}}(\omega) &= 4\text{sinc}^2\left[\frac{t_p(\omega - \omega_0)}{2}\right], \\ R(\omega) &= R_{\text{res}} R_{\text{pulse}}, \end{aligned} \tag{9}$$

where $\kappa_L = \omega_0/Q_L$, $\omega_0/2\pi = 7.203$ GHz, Q_L is the loaded quality factor of the resonator mode and t_p is the fixed duration of the $\pi/2$ and π pulses used in our measurements. We use $Q_i = 117 \times 10^3$ and $Q_L = (Q_i^{-1} + Q_c^{-1})^{-1} = 28.2 \times 10^3$, which are extracted from a fit of VNA measurements of S_{11} at the same power as the pulses used in our measurements. For our experiments $t_p = 10$ μs, which sets the limit to the excitation bandwidth.

In Fig. S6b we compare $A_I^2(\omega)$, $R_{\text{res}}(\omega)$ and $R(\omega)$, which are all normalized. We note that $R_{\text{res}}(\omega)$ and $R(\omega)$ are offset from zero detuning (which we reference to A_I^2) because the measurements were completed at 6.78 mT, whereas the fit gives $B_0 = 6.76$ mT as the center of the transition. The ratio of spins within the $|F, m_f\rangle = |4, -4\rangle \rightarrow |5, -5\rangle$ transition that we excite in our pulsed measurements is then estimated as

$$\beta_b = \frac{\int R(\omega) A_I^2(\omega) d\omega}{\int A_e^2(\omega) d\omega} \approx \frac{1}{54.3}. \tag{10}$$

D. Total number of spins

We are now able to estimate that the total number of spins coupled to the mode of the microwave resonator that contribute to the detected spin echo signals is $N_{\mathrm{tot}} = \beta_b\beta_c\beta_d N_d$. Here, $\beta_d = 1/10$ accounts for the ten equally populated ground states of the bismuth donor spin system at the measurement temperature (400 mK) and magnetic field ($B_0 \sim 6.78$ mT). Therefore:

$$N_{\mathrm{tot}} = \beta_a\beta_b\beta_c\beta_d C_d V_d \approx 9.4 \times 10^3. \tag{11}$$

The definition and numerical values for each of the quantities defining N_{tot} are summarized in Table S3.

TABLE S3. Summary of factors defining N_{tot}.

	Value	Description
β_a	0.6	Donor activation ratio
β_b	1/54.3	Ratio of cavity-pulse bandwidth to total spin linewidth
β_c	0.082	Fraction of resonant spins with the appropriate coupling g_0
β_d	1/10	Fraction of spins at the measured spin transition
C_d	1.03×10^{17} cm^{-3}	Donor concentration
V_d	1009.8 μm^3	Effective donor volume

E. Sensitivity estimate

The sensitivity is defined as the minimum number of spins needed to produce a single spin echo with a SNR of unity. To estimate N_{min}, we determine the single-shot amplitude SNR for a Hahn echo (SNR$_1$) with different applied amplifier gains. We then define $N_{\mathrm{min}} = N_{\mathrm{tot}}/\mathrm{SNR}_1$. The results are displayed in Table S4.

TABLE S4. Single-shot echo SNR and spin sensitivity versus device amplitude gain. Experiments one and two were performed on different days.

	Experiment 1		Experiment 2	
KIPA Amplitude Gain (dB)	SNR$_1$	$N_{\mathrm{min}} \times 10^{-3}$	SNR$_1$	$N_{\mathrm{min}} \times 10^{-3}$
0	0.55(10)	17.1	0.42(0.12)	22.4
2.1	1.91(13)	4.9	1.50(0.16)	6.3
4.4	2.83(32)	3.3	-	-
5.6	-	-	2.61(0.39)	3.6
8.0	3.99(33)	2.4	3.36(0.41)	2.8

Therefore at the highest gain, we are sensitive to approximately 2,800 spins in a single shot measurement. A theoretical estimate of the sensitivity is provided by [7]

$$N_{\mathrm{min}} = \frac{\kappa_L + w}{2 g_0 p}\sqrt{\frac{n_n w \kappa_L}{\kappa(\kappa_L + 2w)}}. \tag{12}$$

where $\kappa = \omega_0/Q_c$ is the coupling rate to the external port, $\kappa_L = \omega_0(Q_i^{-1} + Q_c^{-1})$ is the loaded coupling rate of the resonator, $w^{-1} = T_E$ is the spin echo duration, $p = \tanh\left[\hbar\omega_0/(2k_B T)\right]$ is the spin polarization (where k_B is the Boltzmann constant and T the temperature) and n_n is the number of noise photons added by the detection chain. This equation has been verified in a number of experiments and shown to produce reliable estimates [7, 9, 53]. Using our calculations of N_{min} and measured values for the resonator bandwidth, spin polarization $p = 0.40$, $T_E \approx 20$ μs and $g_0/2\pi = 13.2$ Hz, we can also estimate n_n. With the pump off (no device gain), the first amplifier in the chain is a high-electron-mobility-transistor (HEMT) amplifier, and we calculate

$n_n \approx 14$ photons. Our commercial HEMT amplifier (LNF-LNC0.3_14B) has a specified noise temperature of $T_n = 3.6$ K. Combining this with the estimated 3.5 dB of insertion loss between the HEMT and device (measured at room temperature), we can provide a rough estimate of the system noise that is added to the spin echo signals as $n_n = (1/4 + n_{\rm th})/\eta \approx 12$ photons, where $n_{\rm th} = 1/(2[\exp(\hbar\omega_0/k_B T_n) - 1])$ is the thermal noise per signal quadrature and $\eta = 10^{(-3.5/10)} = 0.45$. At the highest amplitude gain of 8 dB, our extracted $N_{\rm min} = 2,800$ provides an estimated noise of $n_n = 0.22$ photons, whereas the equilibrium noise is expected to be $n_n = 1/4 + n_{\rm th} = 0.6$ photons at $T = 400$ mK. Whilst the extracted noise and expected noise differ slightly, we emphasise that the extracted noise is based on a theoretical model [7] which might not capture all aspects of the experiment. In addition, it may be possible that some parameters in Eq. 12, such as the resonator coupling rates κ and κ_L, vary marginally when performing in-situ amplification. With this in mind, we find the agreement between the values to be satisfactory.

IV. SCALING OF THE SNR WITH AMPLIFIER GAIN

The scaling of the SNR with the gain of the KIPA can be explained using a model based on cavity input-output theory. Here we describe the model and explain how it is used to fit our experimental data.

1. Input-output model

We consider that the KIPA is connected in series with an attenuator with power transmissivity η^2 and a HEMT (Fig. S7). The mode propagating into each component is given by $a_{j,\rm in}$, where $j \in (k, \eta, h)$ is a label for each of the circuit components. Each mode $a_{j,\rm in}$ has the corresponding quadrature operators

$$I_{j,\rm in} = \frac{a_{j,\rm in} + a_{j,\rm in}^\dagger}{2}, \tag{13}$$

$$Q_{j,\rm in} = \frac{a_{j,\rm in} - a_{j,\rm in}^\dagger}{2i}. \tag{14}$$

Each component also has output modes $a_{j,\rm out}$, with quadrature operators equivalent to Eqs. 13 and 14, where the subscripts 'in' are replaced with 'out.' Because the circuit is linear, $a_{k,\rm out} = a_{\eta,\rm in}$, i.e. the mode entering the attenuator is simply the mode that exits the KIPA, and so forth. We also consider bath modes connected to each circuit component, $b_{j,\rm in}$, that add noise to the signal. The bath modes have the associated quadrature operators

$$I_{bj,\rm in} = \frac{b_{j,\rm in} + b_{j,\rm in}^\dagger}{2}, \tag{15}$$

$$Q_{bj,\rm in} = \frac{b_{j,\rm in} - b_{j,\rm in}^\dagger}{2i}. \tag{16}$$

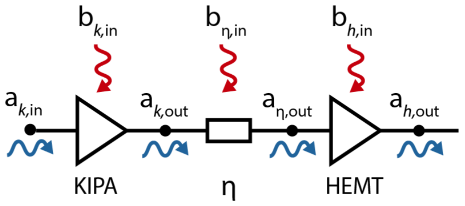

FIG. S7. A model of the measurement system based on cavity input-output theory. The blue arrows label the signal mode as it passes through the three components. The red arrows correspond to bath modes that add noise to the signal.

When amplifying in degenerate mode (i.e. $\omega_p = 2\omega_0$), the input-output relations for the KIPA are given by [15]

$$\begin{pmatrix} I_{k,\text{out}} \\ Q_{k,\text{out}} \end{pmatrix} = A_G \begin{pmatrix} I_{k,\text{in}} \\ Q_{k,\text{in}} \end{pmatrix} + \sqrt{\frac{\gamma}{\kappa}}(A_G + 1) \begin{pmatrix} I_{bk,\text{in}} \\ Q_{bk,\text{in}} \end{pmatrix}, \tag{17}$$

where

$$A_G = \frac{\kappa}{\Delta^2 + (\kappa+\gamma)^2/4 - |\zeta|^2} \begin{pmatrix} (\kappa+\gamma)/2 - |\zeta|\sin(\phi_p) & -|\zeta|\cos(\phi_p) + \Delta \\ -|\zeta|\cos(\phi_p) - \Delta & (\kappa+\gamma)/2 + |\zeta|\sin(\phi_p) \end{pmatrix} - 1. \tag{18}$$

Here, κ is the rate at which the intracavity field is coupled to the measurement port, γ is the rate at which the intracavity field is coupled to internal losses, $\Delta = (\omega_0 - \omega_p/2)$ is the detuning of the pump from twice the cavity's resonant frequency, and ζ is the three-wave mixing strength. For $\Delta = \cos(\phi_p) = 0$, A_G is diagonal. Given that a spin echo signal can be aligned along a single quadrature, we therefore focus on how $I_{k,\text{in}}$ is transformed to $I_{k,\text{out}}$ when $\Delta = 0$ and $\phi_p = 3\pi/2$, which maximizes the gain along the I-quadrature. We find

$$I_{k,\text{out}} = G_k I_{k,\text{in}} + \sqrt{\frac{\gamma}{\kappa}}(G_k + 1) I_{bk,\text{in}} \tag{19}$$

where

$$G_k = \frac{\kappa}{(\kappa+\gamma)/2 - |\zeta|} - 1. \tag{20}$$

To find how the signal is transformed, we take the expectation values of both sides of Eq. 19 where we assume that the bath mode is a thermal state (i.e. $\langle I_{bk,\text{in}} \rangle = 0$), which yields

$$\langle I_{k,\text{out}} \rangle = G_k \langle I_{k,\text{in}} \rangle. \tag{21}$$

To find how the noise is transformed, we calculate the expectation value $\langle I_{k,\text{out}}^2 \rangle$, which is equal to [15]

$$\langle I_{k,\text{out}}^2 \rangle = G_k^2 \langle I_{k,\text{in}}^2 \rangle + (G_k^2 - 1) n_k, \tag{22}$$

where

$$n_k = \frac{\gamma}{\kappa} \frac{G_k + 1}{G_k - 1} \langle I_{bk,\text{in}}^2 \rangle = \frac{\gamma}{\kappa} \frac{G_k + 1}{G_k - 1} \left(\frac{1}{4} + n_{k,\text{th}} \right). \tag{23}$$

n_k is the noise added by the KIPA to the I-quadrature (referred to its input) which has two components, vacuum noise and thermal noise ($n_{k,\text{th}}$).

The input-output relation for the attenuator with amplitude transmissivity η is given by

$$a_{\eta,\text{out}} = \eta a_{\eta,\text{in}} + \sqrt{1-\eta^2}\, b_{\eta,\text{in}}, \tag{24}$$

where

$$\langle I_{b\eta,\text{in}} \rangle = 0, \tag{25}$$

$$\langle I_{b\eta,\text{in}}^2 \rangle = \frac{1}{4} + n_\eta. \tag{26}$$

Here n_η is the noise power for each quadrature of the bath mode measured in excess of vacuum. We therefore see that the attenuator acts as a beamsplitter which mixes in noise with power $\propto 1-\eta^2$.

The HEMT is a non-degenerate amplifier and therefore has input-output relations specified by Caves' Theorem

$$a_{h,\mathrm{out}} = G_h a_{h,\mathrm{in}} + \sqrt{G_h^2 - 1} b_{h,\mathrm{in}}^\dagger, \tag{27}$$

where

$$\langle I_{bh,\mathrm{in}} \rangle = 0, \tag{28}$$

$$\langle I_{bh,\mathrm{in}}^2 \rangle = \frac{1}{4} + n_h. \tag{29}$$

n_h is therefore the noise added by the HEMT in excess of vacuum to each of the quadratures.

Combining Eqns. 19, 24 and 27 we find

$$I_{h,\mathrm{out}} = G_h G_k \eta I_{k,\mathrm{in}} + G_h \sqrt{G_k^2 - 1} \eta I_{bk,\mathrm{in}} + G_h \sqrt{1-\eta^2} I_{b\eta,\mathrm{in}} + \sqrt{G_h^2 - 1} I_{bh,\mathrm{in}}. \tag{30}$$

This leads to the expectation values

$$\langle I_{h,\mathrm{out}} \rangle^2 = G_h^2 G_k^2 \eta^2 \langle I_{k,\mathrm{in}} \rangle^2, \tag{31}$$

$$\langle I_{h,\mathrm{out}}^2 \rangle = G_h^2 G_k^2 \eta^2 \langle I_{k,\mathrm{in}}^2 \rangle + G_h^2 (G_k^2 - 1) \eta^2 n_k + G_h^2 \eta^2 n_{\mathrm{sys}}, \tag{32}$$

where $n_{\mathrm{sys}} = (1/\eta^2 - 1)(1/4 + n_\eta) + (1 - 1/G_h^2)(1/4 + n_h)/\eta^2$ is the 'system noise' referred to the output of the KIPA. These expressions give the square of the signal and the square of the noise measured at the output of the HEMT. They can be referred to the input of the HEMT by dividing by G_h^2. Doing so and taking their ratio, we arrive at an expression for the SNR

$$(\mathrm{SNR})^2 = \frac{G_k^2 \langle I_{k,\mathrm{in}} \rangle^2}{G_k^2 \langle I_{k,\mathrm{in}}^2 \rangle + (G_k^2 - 1) n_k + n_{\mathrm{sys}}}. \tag{33}$$

Eq. 33 tells us that the SNR is limited by three sources of noise: noise on the signal at the input of the KIPA, noise added by the KIPA, and noise added by the components following the KIPA. We should therefore expect the SNR to grow with G_k^2 only so long as the first two contributions, which are amplified by the KIPA, remain smaller than or of a similar magnitude to n_{sys}. Thereafter, both the signal and noise would be amplified by an equivalent amount. We can see this clearly by calculating G_{SNR}^2, which is given by

$$G_{\mathrm{SNR}}^2 = \frac{(\mathrm{SNR})^2}{(\mathrm{SNR})^2|_{G_k^2=1}} = \frac{G_k^2 (\langle I_{k,\mathrm{in}}^2 \rangle + n_{\mathrm{sys}})}{G_k^2 \langle I_{k,\mathrm{in}}^2 \rangle + (G_k^2 - 1) n_k + n_{\mathrm{sys}}}. \tag{34}$$

In the limit $G_k^2 \gg 1$, this simplifies to

$$G_{\mathrm{SNR}}^2 \approx \frac{\langle I_{k,\mathrm{in}}^2 \rangle + n_{\mathrm{sys}}}{\langle I_{k,\mathrm{in}}^2 \rangle + n_k}, \tag{35}$$

where we see that G_{SNR} is constant. Provided $n_{\mathrm{sys}} \gg \langle I_{k,\mathrm{in}}^2 \rangle$, then G_{SNR}^2 is approximately equal to the ratio of the noise added by the components following the KIPA and the noise at the KIPA's input. Therefore, the SNR will be improved by amplifying with the KIPA whenever $n_k < n_{\mathrm{sys}}$.

2. Comparing the model to experiment

In Fig. 4a of the main text we report the SNR of two sets of measurements made with $\phi_p = 0$ as a function of the degenerate amplifier amplitude gain, G_k. To independently match the input-output model to these experiments would require knowledge of all the circuit parameters, including: G_h, η, n_sys, n_k and $\langle I_{k,\mathrm{in}}^2 \rangle$. This can only be achieved via a full analysis of the system noise temperature using a calibrated noise source, which is beyond the scope of the present study. Nevertheless, we can fit the experimental data to a generalized version of Eqn. 33 to confirm that the model is consistent with our measurements. The data in Fig. 4a was fit with the function

$$\mathrm{SNR} = \sqrt{\frac{G_k^2 A}{G_k^2 B + 1}}, \tag{36}$$

where $A = \langle I_{k,\mathrm{in}} \rangle^2/(n_\mathrm{sys} - n_k)$ and $B = (\langle I_{k,\mathrm{in}}^2 \rangle + n_k)/(n_\mathrm{sys} - n_k)$. The fitting parameter B provides an estimate of the ratio of the noise at the input of the KIPA and the system noise, provided $n_\mathrm{sys} \gg n_k$. From the fit we extract $1/B = 22.1$. As explained in Sec. III E, we expect the input noise to the KIPA to be of order one photon (for two quadratures), and the system noise to be of order twenty photons (two quadratures). This result is therefore consistent with our expectations and the sensitivity estimate. We reiterate that this analysis is not intended to serve as a measurement of the noise temperature of the KIPA or the HEMT, but it does provide a reasonable sanity check.

V. SPIN RELAXATION TIME

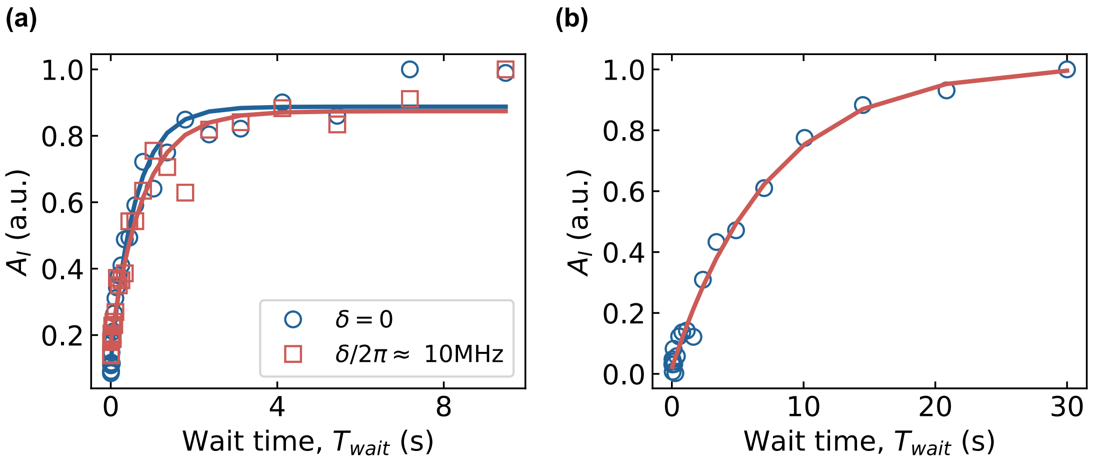

FIG. S8. Measurements of the longitudinal relaxation time T_1 for the electron spins. Integrated echo quadrature signal A_I is plotted as a function of the wait time T_wait after the spin magnetization is initially disturbed by the first pulse of a sequence. The signal is measured with a $N = 200$ pulse CPMG sequence. (a) Inversion recovery experiment performed when spin-cavity detuning δ is zero and when $\delta/2\pi \approx 10$ MHz. Fits with exponential functions reveals $T_{1,\,\mathrm{on}} = 600$ ms and $T_{1,\,\mathrm{off}} = 780$ ms when $\delta = 0$ and $\delta \approx 10$ MHz, respectively. (b) Saturation recovery experiment performed when $\delta = 0$. The exponential fit yields $T_1 = 7.5$ s.

Here we study the longitudinal relaxation processes, which are described by the characteristic time T_1. We again focus on the ESR transition $|F, m_F\rangle = |4, -4\rangle \rightarrow |5, -5\rangle$, which we measure at 6.78 mT.

A known spin relaxation process in microresonator devices occurs due to the Purcell-enhanced spontaneous emission of microwave photons [29, 53]. This process is suppressed quadratically

with the frequency detuning $\delta = \omega_0 - \omega_s$ between the cavity and the spin system according to $\Gamma_p = (\kappa + \gamma)g_0^2/[(\kappa + \gamma)^2/4 + \delta^2]$. To test if our electron spin energy relaxation rate is Purcell limited, we performed an inversion recovery experiment to measure T_1 when spins are on-resonance with the cavity ($\delta = 0$) and when they are detuned ($\delta/2\pi \approx 10$ MHz) during the hold time T_{wait}. The spin-resonator detuning is realized by reducing the DC bias current in the resonator from $I_{\mathrm{DC}} = 3.3$ mA to $I_{\mathrm{DC}} = 2.8$ mA. The spin magnetization is measured after the wait period using a $N = 200$ CPMG pulse sequence with an inter pulse duration of $\tau = 75$ μs. All pulses had a duration of $t_p = 10$ μs. The sequence was repeated 3 times with a repetition of $T_{\mathrm{rep}} = 30$ s. The integrated echo quadrature I signals are presented in Fig. S8a as a function of T_{wait}. The echo decays are well fitted to an exponential function, allowing us to extract the longitudinal relaxation time $T_{1,\,\mathrm{on}} \approx 600(60)$ ms when spins are resonant with the cavity and $T_{1,\,\mathrm{off}} \approx 780(90)$ ms when cavity is detuned from the spins, confirming that the spin energy relaxation rate is not Purcell limited.

To reveal the underlying relaxation mechanism, we perform a saturation recovery experiment with the spins on-resonance with the cavity $\delta = 0$. The sequence commences with a 5 s long saturation pulse, followed by the wait period T_{wait} and spin magnetization measurement using a CPMG pulse sequence. The integrated echo quadrature response A_I and associated exponential fit is presented in Fig. S8b, indicating a $T_1 \approx 7.5$ s. The saturation recovery sequence [1] is known to suppress polarization mixing mechanisms, such as spectral and spatial spin diffusion, by reducing spin polarization gradients in the sample. Such mechanisms are also common in microresonator devices involving donors in silicon [29]. Comparing the values of T_1 obtained in the inversion recovery and saturation recovery measurements, it is clear that the dominant relaxation mechanism for our spin system is spin diffusion.

At low temperatures and magnetic fields, bismuth donors in silicon are expected to have spin T_1 times measuring minutes to hours long when mediated by phonon processes, as confirmed with superconducting microresonators in a previous study [29]. The sub-second spin-diffusion-limited T_1 measured here is therefore many orders of magnitude shorter than the intrinsic time. Spin diffusion is not a typical T_1 process, since energy is simply exchanged between spins in a dipole flip-flop process. However, in a microresonator device that probes only a subset of spins in the sample, it can appear as a T_1 process since the excitations simply diffuse away in the larger spin ensemble. Thus, provided one has enough sample with a spin concentration that ensures sufficient dipole interaction strengths, this could represent a pathway to measuring systems with long intrinsic T_1s at low temperatures.

VI. OPTIMIZING THE SIGNAL-TO-NOISE RATIO

Kinetic inductance is the crucial ingredient in our device that allows the in situ parametric amplification of spin echo signals. However, this inductance does not couple to spins and thus reduces the initial echo signal collected from the donor ensemble. Understanding how the kinetic inductance fraction $\alpha = L_k/(L_k + L_g)$ (where L_k is the kinetic inductance and L_g is the geometric inductance) affects the signal-to-noise ratio and other important parameters, such as the pump power, is therefore important and allows us to identify optimized device parameters.

A. Pump power

We wish to understand the dependence of the pump power needed to produce a specific gain on α. The Hamiltonian for a kinetic inductance parametric amplifier, as utilized in this work, was derived in Ref. 15. In a frame rotating at half of the pump frequency $\omega_p/2$, the Hamiltonian is:

$$H_{\mathrm{kipa}} = \hbar\left(\omega_0 + \delta_{\mathrm{DC}} + \delta_p + K - \frac{\omega_p}{2}\right) a^\dagger a + \frac{\hbar\xi}{2} a^{\dagger 2} + \frac{\hbar\xi^*}{2} a^2 + \frac{\hbar K}{2} a^{\dagger 2} a^2, \tag{37}$$

with the following important Hamiltonian parameters defined as:

$$\delta_{\rm DC} = -\frac{1}{2}\frac{I_{\rm DC}^2}{I_*^2}\omega_0, \tag{38a}$$

$$\delta_p = -\frac{1}{8}\frac{I_p^2}{I_*^2}\omega_0, \tag{38b}$$

$$K = -\frac{3}{8}\frac{\hbar\omega_0}{L_T I_*^2}\omega_0, \tag{38c}$$

$$\xi = -\frac{1}{4}\frac{I_{\rm DC}I_p}{I_*^2}\omega_0 e^{-i\varphi_p}. \tag{38d}$$

This Hamiltonian was derived in the high kinetic inductance limit, where $\alpha \approx 1$. We have re-derived Eq. 38 assuming a non-negligible geometric inductance (i.e. $\alpha < 1$) and find the Hamiltonian to be identical, but with the parameters scaled by the factor α:

$$\delta_{\rm DC} = -\frac{\alpha}{2}\frac{I_{\rm DC}^2}{(I_*[\alpha])^2}\omega_0, \tag{39a}$$

$$\delta_p = -\frac{\alpha}{8}\frac{I_p^2}{(I_*[\alpha])^2}\omega_0, \tag{39b}$$

$$K = -\frac{3\alpha}{8}\frac{\hbar\omega_0}{L_T(I_*[\alpha])^2}\omega_0, \tag{39c}$$

$$\xi = -\frac{\alpha}{4}\frac{I_{\rm DC}I_p}{(I_*[\alpha])^2}\omega_0 e^{-i\varphi_p}. \tag{39d}$$

where once again $\alpha = L_k/L$, $L = L_k + L_g$ and $L_T = lL$ (with l the length of the $\lambda/4$ resonator).

In Eq. 39 we include an implicit dependence of I_* on α, since it is not immediately obvious what impact modifying the kinetic inductance fraction has on I_*. We know that $I_* \propto I_c$ [25], thus equivalently we would like to understand how the critical current of the film I_c varies with α.

The kinetic inductance of a thin rectangular wire varies inversely proportional to its width w and thickness t [20, 56]:

$$L_k \propto \frac{1}{wt}. \tag{40}$$

The thickness dependence can be stronger for very thin films where $t \ll \lambda$ (with λ the penetration depth), but is a suitable approximation for the thickness of NbTiN films considered here (i.e. ≥ 50 nm). To keep our analysis simple, we fix w and only vary the film thickness t to alter L_k. For our thin film ($t \ll w$) the geometric inductance of the coplanar waveguide resonator does not depend (or depends only very weakly) on t [56]. Therefore, by keeping w the same we ensure that the geometric properties (inductance and capacitance) remain constant. The kinetic inductance fraction α thus depends on t as:

$$\alpha = \frac{L_k}{L_k + L_g} = \frac{1}{1 + At}. \tag{41}$$

where $A = L_g/(L_k t)$ and $L_k t$ is a quantity that does not depend on the film thickness (see Eq. 40). This form of dependence has been observed in experiment over a wide range of NbTiN film thicknesses [17].

In general, thin superconducting films display a complicated relationship between critical current and geometry [57]. For thin Nb and NbTi films, it has been observed that the critical current density increases for decreasing film thickness [58, 59], but decays strongly for extremely thin films where $t \ll \lambda$ [59]. Since our film is 50 nm thick and we will be interested in reducing α (i.e. increasing t), we assume that $J_c \propto 1/t$. The critical current is straightforwardly related to J_c via $I_c = J_c(wt)$, therefore we assume I_c and consequently I_* to be independent of the film thickness. The pump strength can now be written explicitly as a function of α as:

$$|\xi| = \frac{\alpha}{4}\frac{I_{\rm DC}I_p}{I_*^2}\omega_0. \tag{42}$$

The DPA gain is determined by the relative strength of the pump $|\xi|$ and the total resonator bandwidth $\kappa_L = \kappa + \gamma$ (with $\kappa = \omega_0 Q_c^{-1}$ and $\gamma = \omega_0 Q_i^{-1}$), as can be seen from the reflection parameter derived using input-output theory [15]:

$$\Gamma(\omega) = \frac{\kappa\kappa_L/2 + i\kappa(\Delta + \omega - \omega_p/2)}{\Delta^2 + \left[\kappa_L/2 + i(\omega - \omega_p/2)\right]^2 - |\xi|^2} - 1. \tag{43}$$

which is written in the laboratory frame. We observe that at half of the pump frequency ($\omega = \omega_p/2$) and for zero detuning ($\Delta = 0$), the gain increases as $|\xi|^2 \to \kappa_L^2/4$.

Assuming that the kinetic inductance fraction α has no impact on the resonator bandwidth κ_L (or that any effect can be compensated for by altering the stepped-impedance filter) and that the resonator frequency ω_0 is kept the same (i.e. by changing the length of the resonator), we can maintain a constant gain for different α by adjusting the pump current, making the following substitution in Eq. 42:

$$I_p \to \frac{I_p}{\alpha}. \tag{44}$$

I.e. a smaller kinetic inductance fraction demands a larger pump current, which means a higher pump power. We assume here that the DC bias current remains the same, set at a value below the critical current of the film.

The impedance of the resonator Z_r also depends on α:

$$\begin{aligned} Z_\mathrm{r} &= \sqrt{\frac{L}{C}}, \\ &= \sqrt{\frac{L_g}{(1-\alpha)C}}, \\ &= \frac{Z_{r0}}{\sqrt{(1-\alpha)}}. \end{aligned} \tag{45}$$

where $Z_{r0} = \sqrt{L_g/C}$ is the resonator impedance in the absence of kinetic inductance.

The pump power is related to the RMS pump current $I_\mathrm{rms} = I_p/\sqrt{2}$ and the resonator impedance according to $P_p = I_\mathrm{rms}^2 Z_r$. Inserting Eqs. 44 and 45, we find the following dependence of P_p on α:

$$P_\mathrm{p} = \frac{I_p^2 Z_{r0}}{2\alpha^2\sqrt{1-\alpha}}. \tag{46}$$

where I_p is the pump current amplitude required in the resonator to achieve a specific level of gain.

Eq. 46 has an optimal point where the pump power is minimized, specifically at $\alpha = 3/4$. This should not be taken too strictly, since for $\alpha \to 1$ ($t \to 0$) the above assumptions (such as the independence of I_* on t) are no longer valid. Reducing the kinetic inductance fraction from 0.8 in the current device by a factor of two to 0.4 requires only a modest increase in the pump power of $\Delta P_p = 10 \log\left[0.8^2\sqrt{1-0.8}/(0.4^2\sqrt{1-0.4})\right] = 3.6$ dB.

B. Signal-to-noise ratio

Next, we consider how the signal-to-noise ratio (SNR) depends on the kinetic inductance fraction α. The SNR is defined by two quantities, the spin sensitivity N_min of the spectrometer and the total number of spins contributing to the echo N_tot [3]:

$$\mathrm{SNR} = \frac{N_\mathrm{tot}}{N_\mathrm{min}}. \tag{47}$$

Of all the parameters in the theoretical expression for N_min (Eq. 12), only g_0 is expected to change with α. The coupling strength $g_0 = \delta B_{1\perp} M \gamma_e$ is determined by the electron spin gyromagnetic ratio γ_e, the spin transition matrix element M and the RMS fluctuations of the magnetic field

$\delta B_{1\perp}$. The RMS magnetic field fluctuations are directly proportional to the current in the resonator $\delta I = \omega_0\sqrt{\hbar/(2Z_r)}$ and hence:

$$\begin{aligned} g_0 &\propto \frac{\omega_0}{\sqrt{Z_r}}, \\ &= \frac{\omega_0(1-\alpha)^{1/4}}{\sqrt{Z_{r0}}}. \end{aligned} \tag{48}$$

where we have used Eq. 45 in the second line. This once again provides an α dependence that assumes we alter the kinetic inductance via the film thickness t, maintaining the same resonator wire width w. We note that Eq. 48 makes the underlying assumption that $\delta B_{1\perp}$ only changes with t through its effect on the impedance, which is generally true so long as $t < w$, λ, d where d is the donor implantation depth (which sets the mean spin-resonator separation), so that the current distribution along the thickness of the film is constant. Finally, we also assume that the resonator frequency ω_0 is kept the same as α is varied by adjusting its length l accordingly.

We arrive at the following α dependence for the spin sensitivity:

$$N_{\text{min}} \propto \frac{1}{(1-\alpha)^{1/4}}. \tag{49}$$

We saw in Sec. III A that the total number of spins is proportional to the effective donor volume $N_{\text{tot}} \propto V_d$, which in turn is proportional to the length of the last $\lambda/4$ sections in the device. Therefore, we expect $N_{\text{tot}} \propto l$.

The frequency of the resonator is given by [15]:

$$\begin{aligned} \omega_0 &= \frac{\pi}{2l\sqrt{LC}}, \\ &= \frac{\pi\sqrt{1-\alpha}}{2l\sqrt{L_g C}}, \\ &= \frac{\pi Z_{r0}\sqrt{1-\alpha}}{2lL_g}. \end{aligned} \tag{50}$$

rearranging we find:

$$l = \frac{\pi Z_{r0}\sqrt{1-\alpha}}{2\omega_0 L_g}. \tag{51}$$

The larger the kinetic inductance fraction, the shorter we must make the resonator to maintain a constant ω_0. This implies that:

$$N_{\text{tot}} \propto \sqrt{1-\alpha}. \tag{52}$$

Finally, putting Eqs. 49 and 52 into Eq. 47 we find:

$$\text{SNR} \propto (1-\alpha)^{3/4}. \tag{53}$$

We reiterate the underlying assumptions in deriving this relation:

- the kinetic inductance fraction α is controlled with the film thickness t
- the geometric inductance L_g remains constant as α is varied
- the frequency ω_0 is kept constant as α is changed by modifying the resonator length l
- the thickness t changes the spin-resonator coupling strength g_0 only through the impedance Z_r

As expected, increasing the kinetic inductance fraction α has a detrimental effect on the SNR, so this should be minimized, whilst balancing the requirement for low/practical pump powers (Eq. 46). For a reduction in α from the current value of 0.8 to 0.4, we would expect a factor 2.3 improvement in the SNR.

Whilst these relations have been found under a number of approximations and assumptions (e.g. $t \ll w$, $t < \lambda$, constant w etc.), improvement in the SNR is likely even for parameter variations outside of these restrictions, though the dependence of P_p and SNR on α should be analyzed further.

C. Sensitivity in the Purcell regime

A useful measure is the absolute sensitivity, which accounts for the finite measurement repetition time, i.e. $N_{\min} \times \sqrt{T_1}$. In our device we found T_1 to be limited by spin diffusion (Sec. V), an effect that depends primarily on the pulse sequence being measured and the spin concentration. However, in superconducting microresonators it is possible to reach the so-called "Purcell regime", where the spin energy relaxation is mediated by emission of microwave photons into the high-Q resonator [29]. Here the spin relaxation time depends on the coupling strength and resonator bandwidth:

$$T_1 = \frac{\kappa_L}{4g_0^2}. \tag{54}$$

for spins in resonance with the resonator. The absolute sensitivity therefore scales as:

$$N_{\min}\sqrt{T_1} \propto \frac{1}{\sqrt{1-\alpha}}. \tag{55}$$

resulting in an absolute SNR (i.e. an SNR that takes into account the repetition time) with the dependence:

$$\frac{\mathrm{SNR}}{\sqrt{T_1}} \propto 1 - \alpha. \tag{56}$$

VII. OPTIMAL RESONATOR DESIGN

An optimal resonator for ESR should concentrate the magnetic mode volume in a well-defined region that is accessible by a sample. As shown in Table S2, the majority of the mode is actually confined in the last quarter-wave segment of the stepped-impedance filter, comprising 54% by mode volume. This implies that we are less than a factor of 2 away from being optimal in the present device. Nevertheless, we can discuss strategies to push the accessible magnetic mode volume even higher.

First, we note that only 19% of the mode is distributed in the rest of the SIF coupling element and the remaining 27% is in the final quarter-wavelength CPW segment that contains the narrow 1 μm wide track (which we refer to as the 'resonator' in the main text). The final segment has the lowest critical current and therefore lowest I_* (which sets the scale of the non-linearity, see Sec. VI) and thus provides the strongest non-linearity in the device for performing amplification. We can vary the relative electrical lengths of the last and second-last (spin detection) segments in the device - making the spin detection region longer will lower the effective non-linearity available and place a greater requirement on the pump strength for amplification. In the extreme case of making the spin detection region twice as long and removing the final 1 μm wide track section all together (effectively creating a half-wavelength resonator), we could expect an accessible magnetic mode volume of up to 81%. As a crude estimate of the effect on the pump power, we assume that the wider 5 μm track in the spin detection region will have an I_* that is 5 times larger than found for the 1 μm track in the current device, and therefore through Eq. 42 will require both the DC (I_{DC}) and pump (I_p) currents to each raise by a factor of 5. This equates to a $5^2 = 25$ times increase in pump power ($\sim$ 14 dB), which is reasonable considering only -50 dBm of pump power is applied in the present measurements.

VIII. THE BOGOLIUBOV AMPLIFIER

Starting from the Hamiltonian of the KIPA in a frame rotating at half the pump frequency (Eq. 37), we introduce a single spin coupled to the mode of the resonator with a strength g_0:

$$\begin{aligned} H_{\mathrm{kipa}} = \hbar\left(\omega_0 + \delta_{\mathrm{DC}} + \delta_p + K - \frac{\omega_p}{2}\right) a^\dagger a + \frac{\hbar\xi}{2} a^{\dagger 2} + \frac{\hbar\xi^*}{2} a^2 + \frac{\hbar K}{2} a^{\dagger 2} a^2 \\ + \frac{\hbar}{2}\left(\omega_s - \frac{\omega_p}{2}\right)\sigma_z + \hbar g_0 (a\sigma_+ + a^\dagger \sigma_-), \end{aligned} \tag{57}$$

where the KIPA-related terms are defined in Eq. 39, ω_s is the Larmor precession frequency of the spin, σ_z is the Pauli Z matrix and σ_+ and σ_- are operators that create and remove an excitation in the spin, respectively.

Defining the detuning parameter as $\Delta = \omega_0 + \delta_{\rm DC} + \delta_p + K - \omega_p/2$, we now consider the detuned regime where $|\Delta| > |\xi| \gg \kappa_L/2$ and Eq. 57 is stable and diagonalizable. Following closely the analysis in Refs. 36, 37, 39, we can diagonalize the Hamiltonian in Eq. 57 by applying the Bogoliubov transformation $U = \exp[r/2(a^2 - a^{\dagger 2})]$, which provides the following Hamiltonian in the Bogoliubov basis:

$$\widetilde{H}_{\rm kipa} = \hbar\delta_a(r)\beta^\dagger\beta + \frac{\hbar}{2}\delta_s\sigma_z + \hbar g_0 \cosh r(\beta^\dagger\sigma_- + \beta\sigma_+) + \hbar g_0 \sinh r(\beta^\dagger\sigma_+ + \beta\sigma_-), \tag{58}$$

where $\delta_a(r) = \Delta/\cosh(2r)$, $\delta_s = \omega_s - \omega_p/2$, $r = \tanh^{-1}(|\xi|/|\Delta|)/2$ is the squeezing parameter and the Bogoliubov operator is given by $\beta = U^\dagger a U = \cosh(r)a - \sinh(r)a^\dagger$. When the systems are in-resonance (i.e. $\delta_a(r) = \delta_s$) and $\delta_a(r) \gg g_0 \sinh r$ so that we can ignore terms that do not conserve energy, the Hamiltonian simplifies to:

$$\widetilde{H}_{\rm kipa} = \hbar\delta_a(r)\beta^\dagger\beta + \frac{\hbar}{2}\delta_s\sigma_z + \hbar g_0 \cosh r(\beta^\dagger\sigma_- + \beta\sigma_+). \tag{59}$$

This describes a Bogoliubov oscillator (i.e. a squeezed oscillator) that is coupled to a spin with a squeezing-enhanced rate $g_0 \cosh r$. This can in principle be used to enhance the signal collected from spins in an ESR experiment, raising the SNR of a measurement. It should, however, be cautioned that the squeezed bath of the Bogoliubov oscillator will also cause dephasing of the spins [39]. However, since the inhomogeneous linewidth of a spin is typically orders of magnitude larger than its coupling strength to the cavity – e.g. even for highly coherent spins in isotopically-enriched ^{28}Si linewidths are of order 2 kHz [60], whilst the spin-resonator coupling strength in the current device is $\sim$ 20 Hz – there may be room for coupling enhancement before squeezing becomes a dominant mechanism for dephasing.

REFERENCES AND NOTES

1. A. Schweiger, G. Jeschke, *Principles of pulse electron paramagnetic resonance* (Oxford Univ. Press, 2001).

2. S. Haroche, J.-M. Raimond, *Exploring the Quantum: Atoms, Cavities and Photons* (Oxford Univ. Press, 2006).

3. J. J. L. Morton, P. Bertet, Storing quantum information in spins and high-sensitivity esr. *J. Magn. Reson.* **287**, 128–139 (2018).

4. N. Abhyankar, A. Agrawal, P. Shrestha, R. Maier, R. D. McMichael, J. Campbell, V. Szalai, Scalable microresonators for room-temperature detection of electron spin resonance from dilute, sub-nanoliter volume solids. *Sci. Adv.* **6**, eabb0620 (2020).

5. Y. Artzi, Y. Yishay, M. Fanciulli, M. Jbara, A. Blank, Superconducting micro-resonators for electron spin resonance-the good, the bad, and the future. *J. Magn. Reson.* **334**, 107102 (2022).

6. T. Yamamoto, K. Inomata, M. Watanabe, K. Matsuba, T. Miyazaki, W. D. Oliver, Y. Nakamura, J. S. Tsai, Flux-driven Josephson parametric amplifier. *Appl. Phys. Lett.* **93**, 042510 (2008).

7. A. Bienfait, J. J. Pla, Y. Kubo, M. Stern, X. Zhou, C. C. Lo, C. D. Weis, T. Schenkel, M. L.W. Thewalt, D. Vion, D. Esteve, B. Julsgaard, K. Mølmer, J. J.L. Morton, P. Bertet, Reaching the quantum limit of sensitivity in electron spin resonance. *Nat. Nanotechnol.* **11**, 253–257 (2016).

8. C. Eichler, A. J. Sigillito, S. A. Lyon, J. R. Petta, Electron spin resonance at the level of 104 spins using low impedance superconducting resonators. *Phys. Rev. Lett.* **118**, 037701 (2017).

9. V. Ranjan, S. Probst, B. Albanese, T. Schenkel, D. Vion, D. Esteve, J. Morton, P. Bertet, Electron spin resonance spectroscopy with femtoliter detection volume. *Appl. Phys. Lett.* **116**, 184002 (2020).

10. Y. Kubo, I. Diniz, C. Grezes, T. Umeda, J. Isoya, H. Sumiya, T. Yamamoto, H. Abe, S. Onoda, T. Ohshima, V. Jacques, A. Dréau, J. F. Roch, A. Auffeves, D. Vion, D. Esteve, P. Bertet, Electron spin resonance detected by a superconducting qubit. *Phy. Rev. B. Condens. Matter Mater. Phys.* **86**, 1–6 (2012).

11. R. P. Budoyo, K. Kakuyanagi, H. Toida, Y. Matsuzaki, S. Saito, Electron spin resonance with up to 20 spin sensitivity measured using a superconducting flux qubit. *Appl. Phys. Lett.* **116**, 194001 (2020).

12. E. I. Rosenthal, C. M. F. Schneider, M. Malnou, Z. Zhao, F. Leditzky, B. J. Chapman, W. Wustmann, X. Ma, D. A. Palken, M. F. Zanner, L. R. Vale, G. C. Hilton, J. Gao, G. Smith, G. Kirchmair, K. W. Lehnert, Efficient and low-backaction quantum measurement using a chip-scale detector. *Phys. Rev. Lett.* **126**, 90503 (2021).

13. F. Lecocq, L. Ranzani, G. A. Peterson, K. Cicak, X. Y. Jin, R. W. Simmonds, J. D. Teufel, J. Aumentado, Efficient qubit measurement with a nonreciprocal microwave amplifier. *Phys. Rev. Lett.* **126**, 020502 (2021).

14. L. Planat, R. Dassonneville, J. Puertas Martínez, F. Foroughi, O. Buisson, W. Hasch-Guichard, C. Naud, R. Vijay, K. Murch, N. Roch, Understanding the saturation power of Josephson parametric amplifiers made from SQUID arrays. *Phys. Rev. Appl.* **11**, 034014 (2019).

15. D. J. Parker, M. Savytskyi, W. Vine, A. Laucht, T. Duty, A. Morello, A. L Grimsmo, J. J. Pla, Degenerate parametric amplification via three-wave mixing using kinetic inductance. *Phys. Rev. Appl.* **17**, 034064 (2022).

16. N. Samkharadze, A. Bruno, P. Scarlino, G. Zheng, D. P. Divincenzo, L. Dicarlo, L. M. K. Vandersypen, High-kinetic-inductance superconducting nanowire resonators for circuit QED in a magnetic field. *Phys. Rev. Appl.* **5**, 1–7 (2016).

17. J. G. Kroll, F. Borsoi, K. L. van der Enden, W. Uilhoorn, D. de Jong, M. Quintero-Pérez, D. J. van Woerkom, A. Bruno, S. R. Plissard, D. Car, E. P. A. M. Bakkers, M. C. Cassidy, L. P. Kouwenhoven, Magnetic-field-resilient superconducting coplanar-waveguide resonators for hybrid circuit quantum electrodynamics experiments. *Phys. Rev. Appl.* **11**, 064053 (2019).

18. N. T. Bronn, Y. Liu, J. B. Hertzberg, A. D. Córcoles, A. A. Houck, J. M. Gambetta, J. M. Chow, Broadband filters for abatement of spontaneous emission in circuit quantum electrodynamics. *Appl. Phys. Lett.* **107**, 172601 (2015).

19. Y. Liu, A. A. Houck, Quantum electrodynamics near a photonic bandgap. *Nat. Phys.* **13**, 48–52 (2017).

20. A. J. Annunziata, D. F. Santavicca, L. Frunzio, G. Catelani, M. J. Rooks, A. Frydman, D. E. Prober, Tunable superconducting nanoinductors. *Nanotechnology* **21**, 445202 (2010).

21. A. T. Asfaw, A. J. Sigillito, A. M. Tyryshkin, T. Schenkel, S. A. Lyon, Multi-frequency spin manipulation using rapidly tunable superconducting coplanar waveguide microresonators. *Appl. Phys. Lett.* **111**, 032601 (2017).

22. Anthony J. Sigillito, Alexei M. Tyryshkin, Thomas Schenkel, Andrew A. Houck, Stephen A. Lyon, All-electric control of donor nuclear spin qubits in silicon. *Nat. Nanotechnol.* **12**, 958–962, (2017).

23. M. R. Vissers, R. P. Erickson, H. S. Ku, L. Vale, X. Wu, G. C. Hilton, D. P. Pappas, Low-noise kinetic inductance traveling-wave amplifier using three-wave mixing. *Appl. Phys. Lett.* **108**, 012601 (2016).

24. D. M. Pozar, *Microwave Engineering* (John Wiley & Sons Inc., ed. 4, 2012).

25. Jonas Zmuidzinas, Superconducting microresonators: Physics and applications. *Annu. Rev. Condens. Matter Phys.* **3**, 169–214 (2012).

26. S. Probst, F. B. Song, P. A. Bushev, A. V. Ustinov, M. Weides, Efficient and robust analysis of complex scattering data under noise in microwave resonators. *Rev. Sci. Instrum.* **86**, 024706 (2015).

27. M. H. Mohammady, G. W. Morley, T. S. Monteiro, Bismuth qubits in silicon: The role of EPR cancellation resonances. *Phys. Rev. Lett.* **105**, 067602 (2010).

28. G. Wolfowicz, A. M. Tyryshkin, R. E. George, H. Riemann, N. V. Abrosimov, P. Becker, H.-J. Pohl, M. L. W. Thewalt, S. A. Lyon, John J. L. Morton, Atomic clock transitions in silicon-based spin qubits. *Nat. Nanotechnol.* **8**, 561–564 (2013).

29. A. Bienfait, J. J. Pla, Y. Kubo, X. Zhou, M. Stern, C. C. Lo, C. D. Weis, T. Schenkel, D. Vion, D. Esteve, J. J.L. Morton, P. Bertet, Controlling spin relaxation with a cavity. *Nature* **531**, 74–77 (2016).

30. J. J. Pla, A. Bienfait, G. Pica, J. Mansir, F. A. Mohiyaddin, Z. Zeng, Y. M. Niquet, A. Morello, T. Schenkel, J. J.L. Morton, P. Bertet, Strain-Induced Spin-Resonance Shifts in Silicon Devices. *Phys. Rev. Applied* **9**, 44014 (2018).

31. M Malnou, MR Vissers, JD Wheeler, J Aumentado, J Hubmayr, JN Ullom, and J Gao. Three-wave mixing kinetic inductance traveling-wave amplifier with near-quantum-limited noise performance. *PRX Quantum* **2**, 010302 (2021).

32. G. A. Rinard, R. W Quine, S. S Eaton, G. R Eaton, W Froncisz, Relative benefits of overcoupled resonators vs inherently low-q resonators for pulsed magnetic resonance. *J. Magn. Reson. Ser. A* **108**, 71–81 (1994).

33. L. J Berliner, S. S Eaton, G. R Eaton. *Distance measurements in biological systems by EPR* (Springer Science & Business Media, 2006), vol. 19.

34. S. Probst, V. Ranjan, Q. Ansel, R. Heeres, B. Albanese, E. Albertinale, D. Vion, D. Esteve, S. J Glaser, D. Sugny, P. Bertet, Shaped pulses for transient compensation in quantum-limited electron spin resonance spectroscopy. *J. Magn. Reson.* **303**, 42–47 (2019).

35. S. Probst, A. Bienfait, P. Campagne-Ibarcq, J. J. Pla, B. Albanese, J. F. Da Silva Barbosa, T. Schenkel, D. Vion, D. Esteve, K. Mølmer, J. J.L. Morton, R. Heeres, P. Bertet, Inductive-detection electron-spin resonance spectroscopy with 65 spins/sqrt(Hz) sensitivity. *Appl. Phys. Lett.* **111**, 10.1063/1.5002540 (2017).

36. A. Metelmann, O. Lanes, T.-Z. Chien, A. McDonald, M. Hatridge, A. A. Clerk, Quantum-limited amplification without instability. arXiv:2208.00024 [quant-ph] (2022).

37. M. Villiers, W. C. Smith, A. Petrescu, A. Borgognoni, M. Delbecq, A. Sarlette, M. Mirrahimi, P. Campagne-Ibarcq, T. Kontos, Z Leghtas,. Dynamically enhancing qubit-oscillator interactions with anti-squeezing. arXiv:2212.04991 [quant-ph] (2022).

38. M. Affolter, W. Ge, B. Bullock, S. C. Burd, K. A. Gilmore, J. F. Lilieholm, A. L. Carter, J. J. Bollinger, Towards improved quantum simulations and sensing with trapped 2d ion crystals via parametric amplification. arXiv:2301.08195 [quant-ph] (2023).

39. I. Shani, E. G Dalla Torre, M. Stern, Coherence properties of a spin in a squeezed resonator. *Phys. Rev. A* **105**, 022617 (2022).

40. M. Malnou, J. Aumentado, M. R. Vissers, J. D. Wheeler, J. Hubmayr, J. N. Ullom, J. Gao, Performance of a kinetic inductance traveling-wave parametric amplifier at 4 kelvin: Toward an alternative to semiconductor amplifiers. *Phys. Rev. Appl.* **17**, 044009 (2022).

41. E. Coronado, Molecular magnetism: From chemical design to spin control in molecules, materials and devices. *Nat. Rev. Mater.* **5**, 87–104 (2020).

42. A. Gaita-Ariño, F. Luis, S. Hill, E. Coronado, Molecular spins for quantum computation. *Nat. Chem.* **11**, 301–309, 2019.

43. A. Cornia, M. Mannini, P. Sainctavit, R. Sessoli, Chemical strategies and characterization tools for the organization of single molecule magnets on surfaces. *Chem. Soc. Rev.* **40**, 3076–3091 (2011).

44. S. Z. Kiss, A. M. Rostas, L. Heidinger, N. Spengler, M. V. Meissner, N. MacKinnon, E. Schleicher, S. Weber, J. G. Korvink, A microwave resonator integrated on a polymer microfluidic chip. *J. Magn. Reson.* **270**, 169–175 (2016).

45. N. Dayan, Y. Ishay, Y. Artzi, D. Cristea, B. Driesschaert, A. Blank, Electron spin resonance microfluidics with subnanoliter liquid samples. *J. Magn. Reson. Open* **2**, 100005 (2020).

46. M. Xu, R. Cheng, Y. Wu, G. Liu, H. X. Tang, Magnetic field-resilient quantum-limited parametric amplifier. arXiv:2209.13652 [quant-ph] (2022).

47. M. Khalifa, J. Salfi, Nonlinearity and parametric amplification of superconducting nanowire resonators in magnetic field. arXiv:2209.14523 [cond-mat.mes-hall] (2022).

48. X. Mi, J. V. Cady, D. M. Zajac, P. W. Deelman, J. R Petta, Strong coupling of a single electron in silicon to a microwave photon. *Science* **355**, 156–158 (2017).

49. K. M. Backes, D. A. Palken, S. Al Kenany, B. M. Brubaker, S. B. Cahn, A. Droster, G. C. Hilton, S. Ghosh, H. Jackson, S. K. Lamoreaux, A. F. Leder, K. W. Lehnert, S. M. Lewis, M. Malnou, R. H. Maruyama, N. M. Rapidis, M. Simanovskaia, S. Singh, D. H. Speller, I. Urdinaran, L. R. Vale, E. C. van Assendelft, K. van Bibber, H. Wang, A quantum enhanced search for dark matter axions. *Nature* **590**, 238–242 (2021).

50. J. J. Wesdorp, F. J. Matute-Caňadas, A. Vaartjes, L. Grünhaupt, T. Laeven, S. Roelofs, L. J. Splitthoff, M. Pita-Vidal, A. Bargerbos, D. J. van Woerkom, P. Krogstrup, L. P. Kouwenhoven, C. K. Andersen, A. Levy Yeyati, B. van Heck, G. de Lange, Microwave spectroscopy of interacting andreev spins. arXiv:2208.11198 [cond-mat.mes-hall] (2022).

51. C. D. Weis, C.C. Lo, V. Lang, A. M. Tyryshkin, R. E. George, K. M. Yu, J. Bokor, S. A. Lyon, J. J. L. Morton, T. Schenkel, Electrical activation and electron spin resonance measurements of implanted bismuth in isotopically enriched silicon-28. *Appl. Phys. Lett.* **100**, 172104 (2012).

52. A. Bruno, G. De Lange, S. Asaad, K. L. Van Der Enden, N. K. Langford, L. Dicarlo, Reducing intrinsic loss in superconducting resonators by surface treatment and deep etching of silicon substrates. *Appl. Phys. Lett.* **106**, 182601 2015.

53. V. Ranjan, S. Probst, B. Albanese, A. Doll, O. Jacquot, E. Flurin, R. Heeres, D. Vion, D. Esteve, J. J.L. Morton, P. Bertet, Pulsed electron spin resonance spectroscopy in the Purcell regime. *J. Magn. Reson.* **310**:106662 2020.

54. J. O'Sullivan, O. W. Kennedy, C. W. Zollitsch, M. Šimenas, C. N. Thomas, L. V. Abdurakhimov, S. Withington, J. J. L. Morton, Spin-Resonance Linewidths of Bismuth Donors in Silicon Coupled to Planar Microresonators. *Phys. Rev. Appl.* **14**, 064050 (2020).

55. V. Ranjan, B. Albanese, E. Albertinale, E. Billaud, D. Flanigan, J. J. Pla, T. Schenkel, D. Vion, D. Esteve, E. Flurin, J. J. L. Morton, Y. M. Niquet, P. Bertet, Spatially resolved decoherence of donor spins in silicon strained by a metallic electrode. *Phys. Rev. X* **11**, 031036 (2021).

56. J. R. Clem, Inductances and attenuation constant for a thin-film superconducting coplanar waveguide resonator. *J. Appl. Phys.* **113**, 013910 (2013).

57. H. L. Hortensius, E. F. C. Driessen, T. M. Klapwijk, K. K. Berggren, J. R. Clem, Critical-current reduction in thin superconducting wires due to current crowding. *Appl. Phys. Lett.* **100**, 182602 (2012).

58. G. Stejic, A. Gurevich, E. Kadyrov, D. Christen, R. Joynt, D. C. Larbalestier, Effect of geometry on the critical currents of thin films. *Phys. Rev. B* **49**, 1274 (1994).

59. N. Pinto, S. J. Rezvani, A. Perali, L. Flammia, M. V. Milošević, M. Fretto, C. Cassiago, N. De Leo, Dimensional crossover and incipient quantum size effects in superconducting niobium nanofilms. *Sci. Rep.* **8**, 4710 (2018).

60. J. T. Muhonen, J. P. Dehollain, A. Laucht, F. E. Hudson, R. Kalra, T. Sekiguchi, K. M. Itoh, D. N. Jamieson, J. C. McCallum, A. S. Dzurak, A. Morello, Storing quantum information for 30 seconds in a nanoelectronic device. *Nat. Nanotechnol.* **9**, 986–991, (2014).
